# Laser speckle particle sizer (SPARSE) informs the size distribution of tissue granularities

**DOI:** 10.1126/sciadv.adu8577

**Published:** 2025-09-26

**Authors:** Zeinab Hajjarian, Nichaluk Leartprapun, Ziqian Zeng, Seemantini K. Nadkarni

**Affiliations:** ^1^ Wellman Center for Photomedicine, Massachusetts General Hospital, Harvard Medical School, Boston, MA 02114 USA.; ^2^ Department of Biomedical Engineering, University of Massachusetts Lowell, Lowell, MA 01854 USA.

## Abstract

Tissues and biomaterials are composed of particles, including cells, nuclei, organelles, protein aggregates, lipid vesicles, and fibers. The size distribution of tissue particles and granularities is altered in various pathologies. These granularities span a wide range from nano- to micrometer scale, posing a unique challenge in quantifying the continuum of particles sizes in intact tissue. We introduce laser Speckle PARticle SizEr (SPARSE), a noncontact optical technique that enables particle sizing over 10 nanometer–to–10 micrometer range, through analyzing the spatiotemporal attributes of polarized laser speckle, back-scattered from biofluids and tissues. We demonstrate that SPARSE effectively quantifies the average particle sizes in milk, blood, and intact tissue without a prior knowledge of particles’ refractive indices or concentrations. Through beam scanning, particle size distribution is mapped in benign and malignant breast tissues with high resolution (~100 micrometer), mirroring histopathological microstructures. By enabling particle sizing in intact biomaterials and tissues, SPARSE holds broad potential for applications across nanomedicine, diagnostics, and biotechnology.

## Teaser

Size distribution of particles within intact tissue is mapped by the noninvasive laser speckle particle sizer (SPARSE).

## INTRODUCTION

Particle size characterization holds broad significance in nanomedicine, biotechnology, and diagnostic pathology (*1*–*6*). In nanomedicine, monitoring the size of nano drugs such as liposomes and lipid-based vesicles in the 50 nm–to–1 μm range are key to predictable and uniform cellular uptake, optimized drug delivery, and the scalability of drug formulations (*2*, *3*). In the food industry, particle size tracking is essential for in-line monitoring of milling, homogenization, and emulsification processes to ensure stability, consistency, and texture of food products (*1*). In blood diagnostics, the size and polydispersity of red blood cells (RBCs) serve to uncover microcyte or macrocyte anemias, thalassemia, sickle cell anemia, hemolysis, and other hematological disorders (*6*–*9*). Similarly, pathological assessment of tumor grade in epithelial neoplasm essentially involves characterizing the enlargement and pleomorphism of nuclei through quantifying the skewed size distributions of nucleus in stained tissue slides (*4*). Furthermore, invasive progression is frequently marked with contrasting size transformations within the extracellular matrix (ECM), namely, reorganization of stromal fibers into larger, aligned bundles (desmoplastic reaction) and their simultaneous breakdown into smaller fragments through the action of matrix metalloproteinases (*10*). These changes highlight the critical role of concurrent size variations at both the cellular and extracellular levels in driving pathological transformations and informing prognosis.

The ubiquitous importance of size characterization has motivated several clinical, industrial, and research-grade techniques. Nevertheless, existing methods fall short in noninvasive, accurate size characterization within intact and optically turbid tissues and biofluids, where particles across a continuum in the nanometer-to-micrometer range intermingle within a richly scattering milieu (*11*). Two of the most common tools for quantifying the size distribution of minute particles are dynamic light scattering (DLS) and laser diffraction (LD) (*12*). DLS evaluates intensity fluctuations of back-scattered light to extract the diffusion coefficient of the particles’ Brownian motion, and in turn their radii over the submicrometer range (*5*). Conversely, LD evaluates much larger particles through angular profiling of diffracted light (*13*). Both techniques, however, rely on dispersing particles in transparent suspensions to conform to single scattering and, therefore, are not amenable for particle sizing in biofluids, intact tissues, or materials with rich optical scattering. A recent technique, florescence membrane imaging, uses a confocal laser scanning microscope to rapidly measure the integrated intensity of fluorescently labeled lipid vesicles and convert it to their size variations in response to fluorescence-based biochemical assays (*14*). Nonetheless, accuracy of these measurements are affected by the confocal depth and photobleaching on measured integrated intensity (*15*). The need to detect micrometer-sized particles in drug formulations, such as protein aggregates and contaminants, has led to the development of backgrounded membrane imaging (BMI) (*16*, *17*). BMI uses vacuum filtration to isolate particles on a membrane, enhancing refractive index contrast, followed by high-throughput imaging and background subtraction for precise size and concentration measurements (*16*). However, it requires drying biofluids and is prone to size underestimation, incomplete imaging, and particle aggregation *(**16*). Another notable approach, angle-resolved low-coherence interferometry (a/LCI) utilizes coherence gating to calculate the angular distribution of light scattered from tissue layers to quantify nuclear size, in the 2.5- to 9-μm range, and enables detection of dysplasia in vivo (*18*, *19*). Nevertheless, its quantitative assessments of particles in submicrometer range remains limited (*20*). Consequently, a technological gap persists for advanced, noncontact techniques to enable mapping of intrinsic particle size distributions over a wide nanometer-to-micrometer range within intact biological specimens and to meet the growing demands of biotechnology and diagnostic medicine.

To address this gap, we introduce a noncontact optical technique, termed laser speckle particle sizer (SPARSE), which characterizes the particles over a range of 10 nm to 10 μm, within intact biomaterials, biofluids, and soft tissues. Unlike existing tools that exploit complex multiangle detection to capture either spatial or temporal variations of single-scattered light, SPARSE is implemented by simply illuminating the sample with a coherent laser beam, making polarization adjustments and capturing the wide-field speckle, diffusely back-scattered from opaque tissue at two orthogonal polarizations via standard complementary metal-oxide semiconductor (CMOS) cameras. When a laser beam is focused on a turbid specimen, the back-scattered light exhibits a diffuse reflectance, i.e., intensity envelope, which is modulated by the time-varying microscale dark and bright intensity grains, termed speckle (*21*, *22*). The distinct spatial intensity envelopes observed in parallel and perpendicular polarized states arise from differences in photon trajectories. Specifically, the size of scattering particles governs the spatial distribution of these trajectories, leading to characteristic differences in the speckle intensity envelope of the two polarization components. These spatial attributes are thus highly sensitive to the size scales of tissue scatterers. Likewise, the temporal attributes of speckle, manifested as time-varying fluctuations in the grainy interference speckle pattern, stem from the dynamic changes in photon path lengths caused by microscale displacements of endogenous scatterers such as nuclei, vesicles, lipid droplets, organelles, and ECM fibers. In fluid samples, where particles freely diffuse, vivid speckle fluctuations are expected. Whereas soft tissue may appear static in conventional sense, rich multiple scattering causes the minute, subwavelength, stochastic thermal motions to create dynamic cumulative optical phase shifts and in turn measurable speckle intensity fluctuations. As with spatial features, the size of scattering particles determines the distinctions of photon path lengths between parallel and perpendicular polarization states. This makes the overall speckle fluctuations rates acutely sensitive to the underlying microstructure and dynamics, while the differential rates between polarization sates specifically reflecting the size of scattering particles (*22*–*25*). The spatiotemporal attributes of polarized laser speckle, thus, may be used to evaluate the size scales of tissue particles. Here, our technical objective is to establish SPARSE as a platform technique that uses the polarimetric analysis of speckle patterns to characterize their spatiotemporal attributes and to quantify a set of metrics that collectively inform the particle size when fed into a size prediction algorithm. The key innovation of SPARSE is the capability to circumvent the constraints of traditional techniques such as DLS, LD, and a/LCI, which require adherence to single-scattering regimen and prior knowledge of particle’s concentrations and their relative refractive indices with respect to the surrounding medium, *n_rel_*, making it applicable to complex specimens of unknown optical absorption and reduced scattering coefficients, μ*_a_* and μ*_s_*′.

Our scientific objective is to demonstrate the accuracy and versatility of SPARSE across multiple applications. We evaluate its performance using a range of increasingly complex specimens encompassing biologically relevant ranges of μ*_a_*, μ*_s_*′, *n_rel_*, and particle sizes, *a*. Each type of specimen presents a unique challenge, allowing us to investigate the advantages, the drawbacks, and the improvement opportunities of this technique. In this regard, polystyrene microspheres serve to test the capacity to perform in-situ sizing of drug delivery carriers without dilution and extractions. Milk phantoms represent challenges existing around inline characterization of nutrient formulations, where opacity and particle size govern texture, stability, and mouthfeel. Whole blood presents the opportunities for hematological analysis and diagnostics within highly scattering and light absorbing biofluids. Last, the prospect of scanning the beam across the sample and mapping the spatially resolved size heterogeneities within freshly excised benign and cancerous lesions of the human breast permits investigating the potential for direct assessment of pathological features, including tumor grade or altered ECM architecture, paving the path for real-time, artefact-free, objective prognosis in freshly excised tissue. By affording simple instrumentation with the powerful capability to perform nondestructive size characterization across a wide size spectrum and diverse optical properties, SPARSE offers substantial potential for industrial and clinical applications, from quality control to diagnostic pathology.

## RESULTS

### Principles of SPARSE

**Fig. 1.**
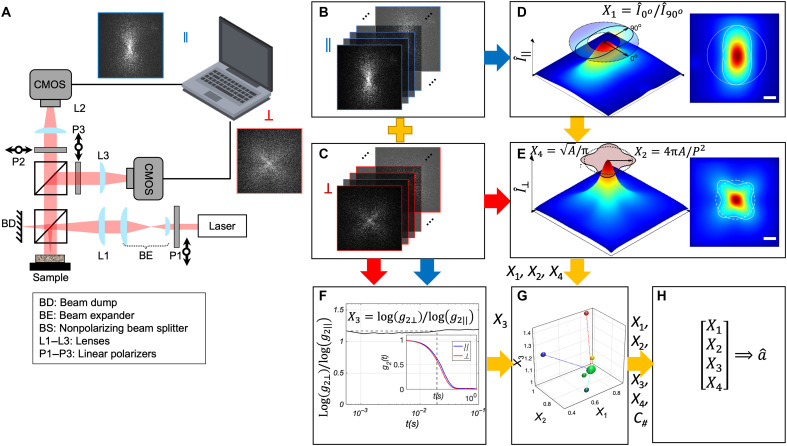
Overview of the SPARSE instrumentation and algorithms. (**A**) Optical setup of SPARSE. A laser beam is linearly polarized and focused on the sample surface. (**B**) Parallel (co-) and (**C**) Perpendicular (cross-) polarized speckle frame series (relative to the illumination polarization axis) emanated from a sample are simultaneously collected by a pair of high-speed CMOS cameras. (**D**) Intensity envelope of the copolarized speckle frame series, ${\hat{I}}_{\|}$. The insets display the top view and the contour of normalized ${\hat{I}}_{\|}$ at 30% of the peak intensity. The long axis of the contour is aligned with the polarization axis. ${\hat{I}}_{\|}$ is radially averaged in the annular region between the inner and outer circles of the contour, yielding an angular profile, from which $X_{1}=\frac{{\hat{I}}_{\|@0{}^{\circ}}}{{\hat{I}}_{\|@90^{{}^{\circ}}}}\;$ is calculated. (**E**) Intensity envelope of the cross-polarized speckle frame series, ${\hat{I}}_{⊥}$. The insets display the top view and the contour of normalized ${\hat{I}}_{⊥}$ at 30% of the peak intensity, from which $X_{2}=\frac{4\pi A}{P^{2}}$ is calculated. Here, *A* and *P* are the contour area and perimeter. Moreover, $X_{4}$ = √(A/π) represents the average radius of the contour. (**F**) Differential decorrelation rates of co- and cross-polarized speckle time series, defined as $X_{3}={\left.\frac{\text{log}\left[g_{2⊥}\left(t\right)\right]}{\text{log}\left[g_{2∥}\left(t\right)\right]}\right|}_{t=t_{\max}}$ is obtained from the intensity autocorrelation, g_2_(*t*), of the speckle time series (inset) at a time point where the vertical separation is the widest. (**G**) To estimate the particle size, the Euclidean distance between experimentally measured [*X*_1_, *X*_2_, *X*_3_, *X*_4_] metrics and the centers of five clusters are computed. The cluster whose center is nearest to the measured metrics is identified. These cluster centers are calculated on the basis of k-means clustering of synthetic [*X*_1_, *X*_2_, *X*_3_, *X*_4_] values simulated for a wide range of particle size and optical property combinations. (**H**) The [*X*_1_, *X*_2_, *X*_3_, *X*_4_] metrics are substituted in the particle size estimation equation, corresponding to nearest cluster, to compute the average particle size. Scale bars, 500 μm.

Biological tissues consist of nanometer- to micrometer-size particles in constant motion due to the exchange between their kinetic energy and the thermal energy of their microenvironment (*23*, *25*). When a coherent, linearly polarized beam is focused on materials or tissues containing intrinsic light-scattering particles (granularities), photons traverse the illuminated volume along different paths, scatter multiple times, and interfere, forming characteristic laser speckle patterns upon reemerging at the surface (*26*–*29*). These laser speckle patterns exhibit distinct spatial intensity envelopes or shapes, with temporal intensity fluctuations of individual speckle grains (Fig. 1).

If granularities in the specimen are smaller than the optical wavelength, λ, most photon trajectories involve only a few wide-angle scattering events that largely preserve the incident polarization, forming the parallel or copolarized component of the speckle pattern (*30*). The time-averaged intensity envelope of copolarized rays, denoted by ${\hat{I}}_{\|}$, exhibits a double-lobed profile aligned with the polarization axis (*29*). A fainter, more dynamic, cross-polarized speckle is contributed by the longer (and less common) paths that traverse a larger number of scattering events. Therefore, when particle size scales are smaller than the optical wavelength, temporal intensity autocorrelation of cross-polarized speckle grains, *g*_2⊥_(*t*) decays faster than copolarized channel, *g*_2||_(*t*), (*31*).

Conversely, when particle size, *a,* approaches or exceeds λ, anisotropic forward scattering contributes to longer photon trajectories with an equal affinity for co- and cross-polarizations (*30*). Consequently, the copolarized intensity envelope, ${\hat{I}}_{\|}$, transforms into a four-leaved pattern, growing a second pair of lobes perpendicular to the polarization axis (Fig. 1) (*29*). Hence, because of equal affinities to the cross-polarized and copolarized channels, differences in the decay rates of *g*_2⊥_(*t*) and *g*_2||_(*t*) are diminished (*32*). In addition, when *a* grows to match and exceed λ, ${\hat{I}}_{\|}$ transforms from dipole to four-leaved. In contrast, the cross-polarized intensity envelope, ${\hat{I}}_{⊥}$ maintains a four-leaved shape, regardless of *a/*λ. However, the ratio of ${\hat{I}}_{⊥}$ area to its perimeter, termed circularity, varies with size. In other words, ${\hat{I}}_{⊥}$ evolves from a roughly circular pattern for a <<λ and a >>λ to a more star-like shape, with increasingly distinguished lobes when *a* approaches λ.

Aside from particle size, the optical properties of tissue also affect both the spatial intensity envelope and temporal dynamics of speckle patterns. Increased particle concentration and refractive index mismatch, *n_rel_ = n_particle_/n_medium_*, foster rich scattering, as quantified by the optical reduced scattering coefficient, μ*_s_*′ (*33*). Since the mean free path is defined as l*=1/μ*_s_*′, increased μ*_s_*′ geometrically scales the optical paths (*34*). Within a given field of view, increasing μ*_s_′* reduces the size of ${\hat{I}}_{\|}$ and ${\hat{I}}_{⊥}$, enhances the contribution from longer photon paths involving many scattering events, gives rise to a four-leaved ${\hat{I}}_{\|}$ pattern, and increases the difference in the decay rates of *g*_2⊥_(*t*) and *g*_2||_(*t*) (*27*, *29*). Conversely, increasing the normalized absorption coefficient, μ*_a_*/μ*_s_′*, i.e., the ratio of absorption and reduced scattering coefficients, prunes the longer paths, pushes the ${\hat{I}}_{\|}$ to the double-lobed pattern, and reduces the difference in the decay rates of *g*_2⊥_(*t*) and *g*_2||_(*t*) (*28*, *29*).

A notably reduced *n_rel_*, which implies weaker scattering, result in less angular deflection and more forward-directed photon paths. Beyond its associations with a generally lower μ*_s_′*, the enhanced forward scattering also alters the spatial distribution of scattered light, specially shifting the radial location of peak intensity of ${\hat{I}}_{⊥}$ away from the centroid toward the middle of the lobes. Although this attribute does not directly report particle size, it qualitatively informs the underlying *n_rel_* of the sample, thus enabling SPARSE to implicitly account for the influence of *n_rel_* variations on other spatiotemporal attributes, without requiring prior knowledge or external input of *n_rel_*.

The theoretical premise of SPARSE is built on these insights and further motivated by the isolated use of these attributes in prior literature. For instance, intensity envelope analysis has been previously used for estimating optical properties (*35*). Earlier work, including ours, has further demonstrated that azimuthal variations in ${\hat{I}}_{\|}$, could be used to semiquantitatively estimate particle sizes using in the 250 nm–to–2.5 μm range (*29*, *36*). Moreover, differences in decay rates of *g*_2⊥_(*t*) and *g*_2||_(*t*) are also documented to qualitatively assess particle sizes in submicrometer range (*30*, *31*).

**Fig. 2.**
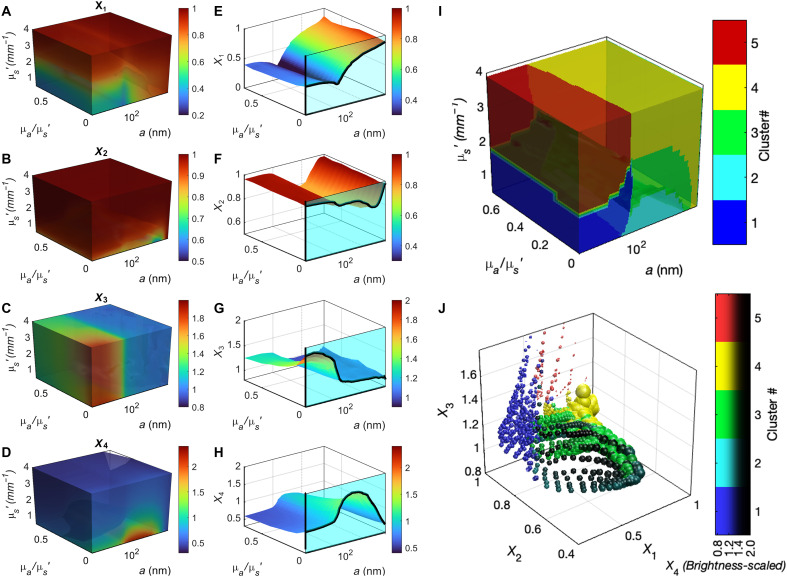
Synthetic library of size-dependent metrics, created by PLCT-MCRT. (**A** to **D**) 3D plots defining the associations between each of the metrics *X*_1_, *X*_2_, *X*_3_, *X*_4_, μ_s_′, and μ*_a_/*μ*_s_*′ for *a*: 10 nm to10 μm. (**E** to **H**) An example of a single cross-sectional plane of the 3D plots (for μ*_s_*′ = 1 mm^−1^ typical of biological tissue) showing the associations between *X*_1_, *X*_2_, *X*_3_, *X*_4_, with particle size and μ*_a_/*μ*_s_*′ variations. The line plots, highlighted by a cyan background corresponds to μ*_s_*′ = 1 mm^−1^ and μ*_a_* = 0. It is evident that *X*_1_ shows a slight decrease as *a* increases from 10 to 100 nm but increases rapidly in the *a*: 125 nm−10 μm range. On the other hand, *X*_2_ is minimized at *a* ~ λ. *X*_3_ presents a decaying trend in *a*, whereas *X*_4_ resembles an inverted U shape, almost reverse of *X*_2_, with a peak that is slightly shifted toward smaller sizes, compared to where *X*_2_ is minimized. The size-dependent variations of *X*_1_, *X*_2_, *X*_3_, *X*_4_ are reduced by absorption, as evidenced by diminished peaks and valleys in the opposite wall plots. (**I**) Clustering analysis of [*X*_1_, *X*_2_, *X*_3_, *X*_4_] metrics partitions the [*a*, μ*_a_*, μ*_s_*′] space to five distinct clusters. (**J**) Scatter plot of the cluster assignments in the [*X*_1_, *X*_2_, *X*_3_, *X*_4_] space. The three axes of the plot correspond to *X*_1_, *X*_2_, and *X*_3_, while *X*_4_ is depicted by varying the adjusted luminescence of the cluster color. The sizes of spherical markers are proportionate to the cube root of particle radii, i.e., $\sqrt[{3}]{a}$. A tailored regression model that formulates *a* as a function of *X*_1_, *X*_2_, *X*_3_, *X*_4_ is obtained for each cluster.

SPARSE leverages the polarimetric analysis of the spatial and temporal attributes of laser speckle to capture these trends via an array of quantitative metrics, denoted as [*X*_1_, *X*_2_, *X*_3_, *X*_4_]. These metrics are illustrated in Figs. 1 and 2, briefly defined below, and elaborated in further details in the Supplementary Materials.

In short, *X*_1_ captures the angular variation of ${\hat{I}}_{\|}$. To this end, normalized ${\hat{I}}_{\|}$ is contoured at 30% of its peak intensity. From the coordinates of the individual points on the contour, its centroid is determined. Subsequently, the inner and outer circles of the contour are drawn to have their centers at the centroid and their perimeter passing through the points on the contour that are closest and farthest from the centroid, respectively. Subsequently, ${\hat{I}}_{\|}$ is radially averaged in the annular region between the inner and outer circles of the contour, yielding an angular profile, from which $X_{1}=\frac{{\hat{I}}_{\|@0{}^{\circ}}}{{\hat{I}}_{\|@90{}^{\circ}}}\;$ is calculated. Here, ${\hat{I}}_{\|@0{}^{\circ}}$ and ${\hat{I}}_{\|@90{}^{\circ}}$ represent the radially averaged ${\hat{I}}_{\|}$ at 0° and 90^o^ with respect to horizontal axis in the annular region between inner and outer circles of the contour. The circularity of ${\hat{I}}_{⊥}$ is quantified by $X_{2}=\frac{4\mathrm{\pi A}}{P^{2}}$, where *A* and *P* are the area and the perimeter of the ${\hat{I}}_{⊥}$ contour at 30% of its peak intensity. The 30% threshold is selected as a consistent and practical criterion to define the contours of ${\hat{I}}_{\|}$ and ${\hat{I}}_{⊥}$ as it reliably captures the spatial extent of the multiply scattered region, roughly one l* away from the illumination point, while avoiding dominance by ballistic light or background noise (*35*). To capture the temporal features, the differential decorrelation rate of *g*_2⊥_(*t*) and *g*_2||_(*t*) is calculated by $X_{3}={\left.\frac{\text{log}\left[g_{2⊥}\left(t\right)\right]}{\text{log}\left[g_{2∥}\left(t\right)\right]}\right|}_{t=t_{\max}}$ at *t = t_max_*, where the slopes of the two curves and in turn their vertical separation is maximized*.* Last, the radial extent of ${\hat{I}}_{⊥}$ is captured by *X*_4_* = √*(*A/*π)*.* These metrics are then incorporated within a prediction algorithm (described below) to estimate the scattering particle size, *a* (Fig. 1). Together, the *X*_1_–*X*_4_ metrics quantify the most prominent variations in polarized laser speckle attributes with respect to scattering particle size. In what follows, we detail how SPARSE leverages the full extent of the spatiotemporal speckle attributes through integrating previously isolated features into a unified framework to quantitatively assess particle size, across three orders of magnitude, 10 nm to 10 μm, in biological samples, independent of their optical properties.

### SPARSE particle size estimation algorithm

In parallel to experimental methods for quantifying the [*X*_1_, *X*_2_, *X*_3_, *X*_4_] metrics, SPARSE further requires a systematic and quantitative examination of how [*X*_1_, *X*_2_, *X*_3_, *X*_4_] change with particle size and optical properties, beyond qualitative speculations, trends reported in the literature, and experimental evidence described above. This is imperative for building the basis for an accurate and robust particle-sizing estimation model. To develop the SPARSE particle size estimation algorithm, we expanded our previously developed polarized-light Monte Carlo ray tracing approach to simulate both the diffused light intensity and its correlation-transfer to obtain not only the first-order (i.e. average spatial intensity) but also the second-order (i.e. temporal intensity auto-correlation) statistics of both co- and cross- polarized speckle (*28*, *29*). This permitted simulation of both the polarized intensity envelopes ${\hat{I}}_{\|}$ and ${\hat{I}}_{⊥}$ and polarized intensity temporal autocorrelation curves *g*_2||_(*t*) and *g*_2⊥_(*t*) in turbid media of biologically relevant optical properties. Specifically, μ*_s_′* was varied in the 0.25 to 4 mm^−1^ to cover the range reported in the literature for most biological tissues and biofluids (*34*, *37*–*39*). Moreover, μ*_a_* was changed in the 0 to 70% μ*_s_′* range (*31*). While light propagation may be treated as diffusion only when μ*_a_* < 30% μ*_s_′*, using ray tracing for modeling light transport in tissue allowed us to stretch the range of acceptable μ*_a_* values further before excess absorption prohibited the detection of spatiotemporal attributes (*31*, *39*). Last, particle size was varied in the *a* = 10 nm–to–10 μm range (see the Supplementary Materials) (*39*). The first iteration of simulations was conducted assuming the average *n_rel_ = 1.1* reported for biological tissues, and μ*_s_′* variations were achieved solely by modifying the particle size and concentration and not the *n_rel_* (*37*, *40*, *41*)*.* In addition, multiple iterations were repeated by considering other biologically relevant *n_rel_* to account for the influence of refractive index variations (figs. S1 to S4) (*37*, *40*, *41*). We then reconstructed the ${\hat{I}}_{\|}$, ${\hat{I}}_{⊥}$, *g*_2||_(*t*), and *g*_2⊥_(*t*) from the simulated photon trajectories as detailed in the Supplementary Materials. From the simulated polarized speckle attributes, a synthetic library of [*X*_1_, *X*_2_, *X*_3_, *X*_4_] metrics was generated across a broad range of (*a*, μ*_a_*, and μ*_s_′*) and multiple *n_rel_* values in the 1.03 to 1.2 range (*37*, *40*, *41*).

As an example, for μ*_s_ =* 1 mm^−1^ and μ*_a_* = 0, our simulation results provide a detailed account of ${\hat{I}}_{\|}$, and in turn *X*_1_ variations with size, demonstrating that when *a* ~ 10s nm, ${\hat{I}}_{\|}$ is almost elliptical with a subtle notch in the midline that deepens as *a* increases and approaches 100 nm, causing *X*_1_ to decrease with *a* in this range. A second pair of lobes raise above 125 nm, leading to the growth of *X*_1_ with *a,* before it saturates beyond 10 μm (Fig. 2E, line plots). Our results also show that *X*_2_ remains close to 1, for sizes much smaller or larger than λ, but reduces substantially near λ(Fig. 2F, line plot). While *X*_2_ is minimized in the vicinity of *a* = λ, our simulations indicate that the precise location of the minimum also likely varies with *n_rel_*. *X*_3_ is consistently decreasing with *a* but exhibits a substantially steeper slope in submicrometer range (Fig. 2G, line plot). *X*_4_ presents an inverted U trend with *a*, almost opposite to *X*_2_ behavior. However, the location at which *X*_4_ is maximized is slightly shifted toward smaller sizes, compared to the absolute minimum of *X*_2_ near *a* = λ. The line plots detailed above correspond to μ*_s_′ =* 1 mm^−1^ and μ*_a_* = 0. Similar line plots may be obtained by referring to cross sections of Fig. 2 (A to D), at the intersection of the desired μ*_s_′* and μ*_a_* combination. These line plots exhibit different increasing or declining trends for [*X*_1_, *X*_2_, *X*_3_, *X*_4_] with particle radii, and the specific location and magnitude of the maxima and minima are additionally modulated by μ*_a_* and μ*_s_′*. For instance, increasing the μ*_a_/*μ*_s_* tends to diminish the [*X*_1_, *X*_2_, *X*_3_, *X*_4_] variations with *a*, as evidenced by cross sections parallel to the cyan-filled wall nearing the opposite wall.

Therefore, the insight from Monte-Carlo ray tracing (MCRT) drives the selection of *X*_1_ to *X*_4_ by analyzing the spatiotemporal attributes of the polarized laser speckle. These metrics are designed to quantify the most salient variations in polarized speckle with the scattering particle size. Collectively, they serve as quantitative predictors of particle size across three orders of magnitude while also implicitly accounting for μ*_a_* and μ*_s_′* variations, as depicted in Fig. 2. MCRT simulations further identify an additional attribute, the location of the radial coordinate of maximum intensity of ${\hat{I}}_{⊥}$, which informs the *n_rel_*. They further demonstrated that the variations in *n_rel_* primarily affect [*X*_1_, *X*_2_, *X*_3_, *X*_4_] metrics for particle sizes *a* > 500 nm, with the most pronounced effects observed at markedly lower *n_rel_* values (figs. S1 and S2). Together, these considerations make the SPARSE framework applicable to complex specimens of unknown μ*_a_* and μ*_s_*′ and *n_rel_*.

From the above trends, it is evident that the mathematical forms relating [*X*_1_, *X*_2_, *X*_3_, *X*_4_] metrics to *a* vary both across the size range and in response to optical properties, suggesting that multiple particle size estimation equation may be needed within different regions of [*a*, μ*_a_*, μ*_s_′*] and in turn [*X*_1_, *X*_2_, *X*_3_, *X*_4_] space, and for different *n_rel_* values. Cluster analysis of synthetic [*X*_1_, *X*_2_, *X*_3_, *X*_4_] metrics, obtained for each *n_rel_* value, reveals that this space may be divided to an optimum number of five clusters. Subsequently, a step-wise regression analysis of [*X*_1_, *X*_2_, *X*_3_, *X*_4_] within individual clusters yields a tailored particle size estimation equation expressing *a* as a function of [*X*_1_, *X*_2_, *X*_3_, *X*_4_] belonging to that cluster (Eqs. S10 to S14).

Therefore, to estimate the particle size from experimentally measured polarized speckle frame series, we simply need to follow the steps outlined in the flowchart of Fig. 1 to calculate the experimental [*X*_1_, *X*_2_, *X*_3_, *X*_4_] metrics, identify the cluster to which the sample belongs, plug in the metrics in the corresponding particle size estimation equation (Eqs. S10 to S14), and obtain *a*. In this respect, *n_rel_* variations may be readily inferred and catered to by inspecting the ${\hat{I}}_{⊥}$ to assess the radial distance of the peak intensity, prompting the SPARSE to use a modified particle size estimation model. Details of the clustering analysis and the particle size estimation equations are elaborated in Materials and Methods and the Supplementary Materials. In what follows, we investigate and validate the SPARSE approach by applying it for particle size measurement in monodispersed suspensions and biological tissues of increasing complexities.

### Characterizing the polystyrene microsphere suspensions

**Fig. 3.**
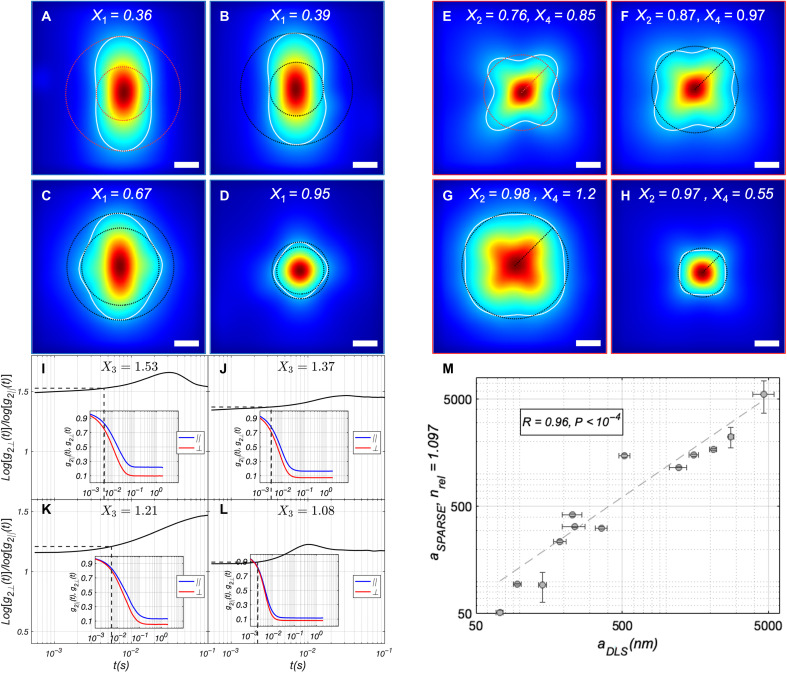
Sizing polystyrene microspheres. (**A** to **D**) ${\hat{I}}_{\|}$ corresponding to aqueous glycerol suspensions of polystyrene microspheres exhibiting radii of 75, 100, 250, and 5000 nm, respectively. Also displayed in each panel is the contour of ${\hat{I}}_{\|}$ at 30% of its maximum together with the calculated *X*_1_ values. (**E** to **H**) Corresponding ${\hat{I}}_{⊥}$ of polystyrene microsphere phantoms. Similarly, the contour at 30% is traced and the calculated *X*_2_ and *X*_4_ are displayed. (**I** to **L**) Ratio of the speckle decorrelation rate in perpendicular and parallel polarization, defined as log[*g*_2⊥_(*t*)]/log[*g*_2||_(*t*)]. The inset displays *g*_2⊥_(*t*) in red and *g*_2||_(*t*) in blue. *X*_3_ is calculated at the temporal point where the *g*_2_(*t*) slope is maximized (dashed lines in the main plot and inset). (**M**) Scatter diagram of particle radius, *a*, predicted by SPARSE exhibits a strong, statistically significant correlation with standard DLS measurements (*R* = 0.96, *P* < 0.0001). Scale bars, 500 μm.

We first evaluated monodispersed polystyrene microsphere suspensions in aqueous glycerol solutions to assess the accuracy of the SPARSE approach to measure *a* in the 50 nm–to–5 μm range, as illustrated in Fig. 3.

Microspheres in 50- to 400-nm and 500- to 5000-nm ranges were suspended in 90% (*n*_μ*sphere*_ = 1.59, *n_medium_* = 1.45, *n_rel_* = 1.097) and 70% (*n_μsphere_* = 1.59, *n_medium_* = 1.44, *n_rel_* = 1.113) aqueous glycerol mixtures, respectively. In addition, the full range of microspheres were further dispersed in 60% glycerol-water mixtures, exhibiting *n_rel_* = 1.12. These conditions span a representative biologically relevant range of approximately n_rel_ ≈ 1.09 to 1.12 (*42*, *43*). Figure 3 shows ${\hat{I}}_{\|}$, ${\hat{I}}_{⊥}$, g_2||_(*t*), and g_2⊥_(*t*) obtained experimentally using the SPARSE instrument (Fig. 1A) for a selected subset of polystyrene microsphere suspensions with *a* of 75, 100, 250, and 5 μm (μ*_s_*′ was 0.77, 1.08, 0.97, and 0.63 mm^−1^, respectively).

As *a* increases, *X*_1_ increases from 0.36 to 0.95, while *X*_2_ shows less variation at these selected sizes, except for the lower value at 75 nm. *X*_3_ and *X*_4_ exhibit the expected decreasing and concave trends, respectively (similarly observed in synthetic data of Fig. 2). SPARSE holds the promise to estimate *a* regardless of μ*_s_*′ variations.

The experimentally determined [*X*_1_, *X*_2_, *X*_3_, *X*_4_] metrics of polystyrene bead are compared to the cluster centers to identify the one with the shortest Euclidean distance. Substituting the [*X*_1_, *X*_2_, *X*_3_, *X*_4_] metrics in the particle size estimation equation of corresponding clusters yields the predicted *a* value. A statistically significant correlation is observed between SPARSE and DLS measurements (*R* = 0.96, *P* < 10^−4^) across the entire 50 nm–to–5 μm polystyrene bead sizes. Also noted in the scatter plot is the deviation of SPARSE from DLS for *a* < 100 nm, which is likely caused by the substantial dependence of particle size estimation equation on *X*_3_ in this size range and in turn the critical modulation of this metric by both *a* and μ*_s_*′, at the scale of few tens of nanometer, as discussed later. We have summarized SPARSE and DLS measurement results for all the polystyrene microspheres, along with manufacturer-provided nominal sizes, and the theoretically calculated μ*_s_*′ values in table S3. Similar agreements are observed between SPARSE measurements and nominal particle sizes between SPARSE and DLS measurements for microspheres suspended in 60% aqueous glycerol and across SPARSE measurements of microspheres suspended in glycerol solutions with varying *n_rel_* values (fig. S5).

### Characterizing the particle size in milk specimens of varying fat content

**Fig. 4.**
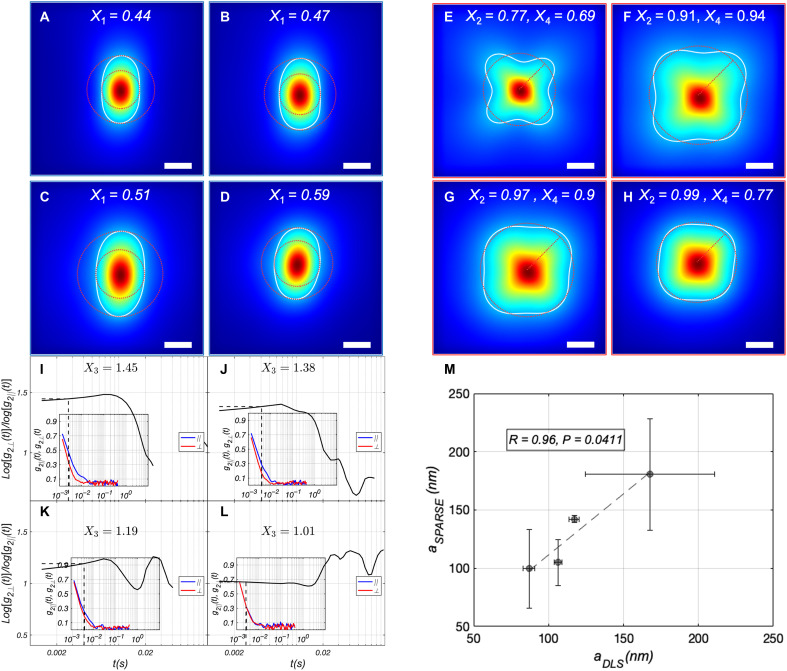
Characterizing the particle size in milk. ${\hat{I}}_{\|}$ of (**A**) nonfat, (**B**) low-fat (1%), (**C**) reduced fat (2%), and (**D**) whole milk (4%), respectively. Also displayed in each panel is the contour of ${\hat{I}}_{\|}$ at 30% of its maximum together with the calculated *X*_1_ values. (**E** to **H**) Corresponding ${\hat{I}}_{⊥}$ of milk specimens. Similarly, the contour at 30% is traced and the calculated *X*_2_ and *X*_4_ are displayed. (**I** to **L**) Ratio of the speckle decorrelation rate in perpendicular and parallel polarization, defined as log[g_2⊥_(*t*)]/log[g_2||_(*t*)]. The inset displays *g*_2⊥_(*t*) in red and *g*_2||_(*t*) in blue. *X*_3_ is calculated at the temporal point where the *g*_2_(*t*) slope is maximized, as indicated by a dashed line in both the main plot and the inset. (**M**) Scatter diagram of *a*, predicted by SPARSE exhibits a strong, statistically significant correlation with standard DLS measurements (*R* = 0.96, *P* = 0.0411). Scale bars, 500 μm.

Particle sizing of milk is an integral part of quality control in the dairy industry (*1*). It has been reported that in homogenized milk, proteins like casein micelles exhibit typical radii of 100 nm, while fat globules span the 200 nm–to–2 μm range (*44*). The reported values for the refractive indices of fat globules (*n_f_* = 1.46, *n_f_/n_w_* = 1.1) and protein micelles (*n_p_ = 1.57*, *n_p_/n_w_* = 1.18) are either comparable or higher than those for polystyrene microsphere phantoms (*45*, *46*). Here, *n_w_* = 1.33 represents the refractive index of water, which constitutes most milk content. We tested the utility of SPARSE for particle sizing in commercially available nonfat (0%), low-fat (1%), reduced-fat (2%), and whole milk (4%), with unknown optical properties and refractive indices (Fig. 4).

Our measurements indicate that by increasing fat content from 0 to 4%, *X*_1_ increases from 0.44 to 0.59, *X*_2_ increases from 0.77 to 0.99, and *X*_3_ consistently decreases from 1.45 to 1.01. *X*_4_ increases from 0.69 in 0% fat milk to 0.94 in 1% milk but then reduces to 0.9 and 0.77 in 2 and 4% fat milk, respectively. This is likely due to the competing effects of simultaneous increase in *a* and μ*_s_*′ with fat content, in respectively raising and reducing *X*_4_, in this range.

On the basis of these metrics, SPARSE estimates average particle sizes of 99.6, 105, 142, and 180 nm for 0, 1, 2, and 4% milk samples, which strongly and significantly correlate with DLS measurements (*R* = 0.96, *P* = 0.0411). Deviations are larger for 1 and 2% fat milk, likely because size differences are in the order of few tens of nanometer, approaching the resolution limit of SPARSE and DLS (*47*). These estimates also align with the expected trend in particle sizes based on the proportions of protein micelles and fat globules, suggesting that SPARSE’s prediction algorithm remains applicable to these biologically relevant samples (*45*).

### Tracing the shrinkage of RBCs in whole blood samples of increased tonicity

Sizing RBCs is essential for diagnosing a variety of anemias and other blood disorders that result in microstructural changes in RBCs (*7*). Here, we tested the capability of SPARSE to monitor the changes in RBC size, in response to variations in plasma tonicity, within fresh whole-blood. Unlike microspheres and milk, which exhibit negligible absorption at λ = 633 nm, blood demonstrates both scattering and absorption due to its hemoglobin content, with μ*_s_*′ = 1.2 mm^−1^ and μ*_a_ =* 0.3 mm^−1^ (*48*). Furthermore, in whole blood, RBCs (*n*_RBC_ ≈ 1.402) suspended in plasma (*n*_plasma_ ≈ 1.36) elicit an index mismatch of *n_rel_* ≈ 1.03. By spiking the blood with saline solutions of varying NaCl concentrations, we induce osmotic stress that modulates RBC size, μ*_a_*, μ*_s_*′, and *n_rel_*. This presents an opportunity to evaluate the feasibility of applying SPARSE to track changes in particle size, alongside concurrent alterations in optical properties within biological samples.

Normal whole swine blood samples were obtained from a commercial vendor (Animal Technologies, USA). The samples were aliquoted, and the tonicity of each aliquots was modified by spiking 50 μl of blood with 10 μl of saline solutions containing 0.9 to 8% NaCl, yielding final NaCl concentrations from 0.9 to 2.1% (*49*). Increased osmolarity caused RBC shrinkage, likely enhancing the *n_rel_* and, in turn, μ*_s_*′ (*50*). Simultaneously, conformations changes in heme groups within RBC reduced μ*_a_* at the illumination wavelength (*49*, *51*). This is visually evident from the brighter red hues observed in samples with higher NaCl concentrations (fig. S7). Compared to humans (RBC sphere-equivalent radius: 2.67 to 2.88 μm), RBCs tend to be smaller in swine (sphere-equivalent radius: 2.3 to 2.6 μm) (*52*). They are also less resilient to osmotic shock and may undergo hemolysis (*53*).

**Fig. 5.**
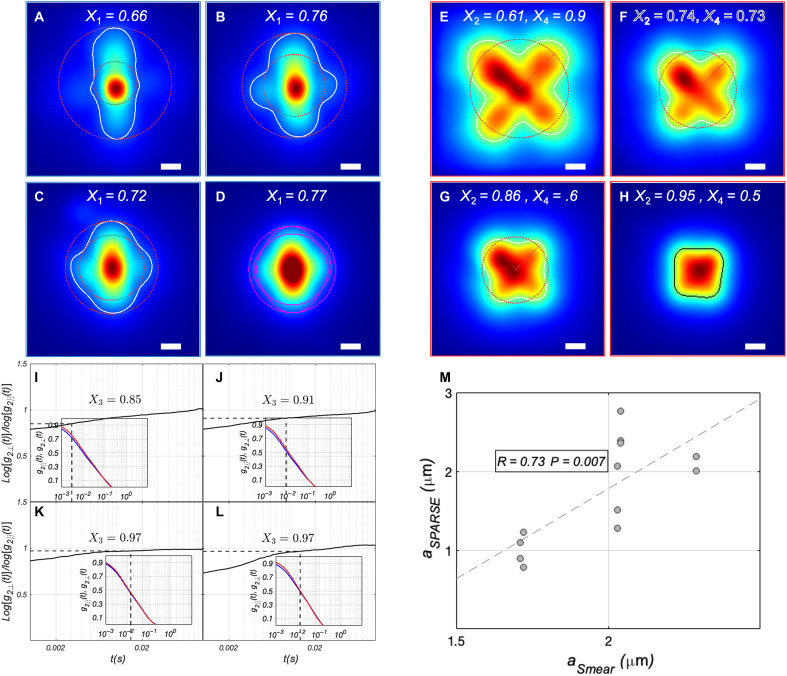
Tracing RBC shrinkage in response to increased saline concentration of plasma. ${\hat{I}}_{\|}$ of whole blood samples of (**A**) 0.9%, (**B**) 1.08%, (**C**) 1.42%, and (**D**) 1.75% NaCl concentration, respectively. Also displayed in each panel is the contour of ${\hat{I}}_{\|}$ at 30% of its maximum together with the calculated *X*_1_ values. (**E** to **H**) Corresponding ${\hat{I}}_{⊥}$ of whole blood samples. Similarly, the contour at 30% is traced and the calculated *X*_2_ and *X*_4_ are displayed. (**I** to **L**) Ratio of the speckle decorrelation rate in perpendicular and parallel polarization, defined as log[*g*_2⊥_(*t*)]/log[*g*_2||_(*t*)]. The inset displays *g*_2⊥_(*t*) in red and *g*_2||_(*t*) in blue. *X*_3_ is calculated at the temporal point where the *g*_2_(*t*) slopes are maximized, as marked by the dashed line in the main graph and the inset. (**M**) Scatter diagram of *a*, predicted by SPARSE exhibits a strong, statistically significant correlation with RBC size obtained from stained smear microscopy (*R* = 0.73, *P* = 0.007). Scale bars, 500 μm.

Figure 5 depicts ${\hat{I}}_{\|}$, ${\hat{I}}_{⊥}$, *g*_2||_(*t*), and *g*_2⊥_(*t*) for swine blood samples of 0.9, 1.08, 1.42, and 1.75% final NaCl concentration. The ${\hat{I}}_{\|}$ of isotonic blood appears elliptical, with X_1_ = 0.66, which is lower compared to polystyrene phantoms of comparable radii (swine RBC radius ~2.3 to 2.6 μm) (*54*). Our MCRT simulations suggest that this deviation arises from the considerable absorption of blood at 633 nm, where μ*_a_* > 0.3 mm^−1^ (Supplementary Materials) (*48*). In addition, unlike polystyrene phantoms and milk, at lower NaCl concentrations, the peak of ${\hat{I}}_{⊥}$ occurs in the middle of leaflets rather than the centroid. Our MCRT simulations attribute this to the lower *n_rel_* = 1.03 of whole blood (figs. S1, S2, and S8) (*55*). This marked *n_rel_* reduction at 0.9 and 1.08% is inferred from ${\hat{I}}_{⊥}$ and triggers SPARSE to switch to the particle size estimation equations developed for *n_rel_* = 1.03 (Supplementary Methods). This model is used whenever the radial distance of ${\hat{I}}_{⊥}$ maxima from the centroid exceeds 50% of *X*_4_. As saline concentration increases, secondary lobes emerge in ${\hat{I}}_{\|}$ and *X*_1_ raises to 0.77. In addition, ${\hat{I}}_{⊥}$ contracts, and its peak intensity shifts from the middle of leaflets to the centroid. This results in increased *X*_2_ and decreased *X*_4_. The trends in ${\hat{I}}_{\|}$, ${\hat{I}}_{⊥}$, *X*_1_, *X*_2_ and *X*_4_ reflect the contraction of speckle intensity envelopes, suggesting a decrease in μ*_a_*, an increase in *n_rel_*, and a corresponding increase in μ*_s_*′. As expected, *X*_3_ remains close to unity. However, both *g*_2||_(*t*), and *g*_2⊥_(*t*) decay faster at higher NaCl concentrations, reflecting a combination of size reduction, reduced absorption, and enhanced scattering (*28*).

Metrics [*X*_1_, *X*_2_, *X*_3_, *X*_4_] were used to identify the closets matching clusters and were then substituted in the corresponding particle size estimation equation to obtain *a*. To validate the absolute accuracy of SPARSE, the estimated RBC sizes were compared to measurements from the standard stained blood smear microscopy (fig. S9). Visual inspection of digitized slides revealed a progressive reduction in RBCs size with increasing NaCl concentrations. At higher concentrations (≥1.75%), ghost cells began to appear, indicating membrane rupture and lysis. Digitized smear images were processed with a custom MATLAB script to segment RBCs, calculate equivalent radii, and generate size distributions with corresponding means and SDs (fig. S9). At a NaCl concentration of 0.9%, SPARSE estimated *a* = 2.2 μm, closely matching both smear microscopy (*a* = 2.29 μm) and the literature-reported value of 2.3 μm for swine RBCs (*54*). At 2.1% NaCl, SPARSE reported a markedly reduced size of *a* = 1.00 ± 0.14, whereas smear microscopy measured *a* = 1.72 ± 0.19 μm (table S4). While both techniques consistently captured the progressive decrease in RBC size with increasing saline concentration, smear microscopy exhibited lower sensitivity to subtle size variations compared to SPARSE, as discussed later. Figure 5M presents a scatter plot comparing RBC size measured by SPARSE with those obtained from smear microscopy image analysis. A strong, statistically significant correlation was observed between the two measurements (*R* = 0.73, *P* = 0.007). Collectively, these findings affirm the capability of SPARSE to measure physiologically consistent, absolute size measurements in whole blood, capturing subtle changes in RBC morphology that often elude conventional light scattering techniques such as DLS.

### Mapping particle size distributions in heterogenous benign and cancerous tissue specimens

**Fig. 6.**
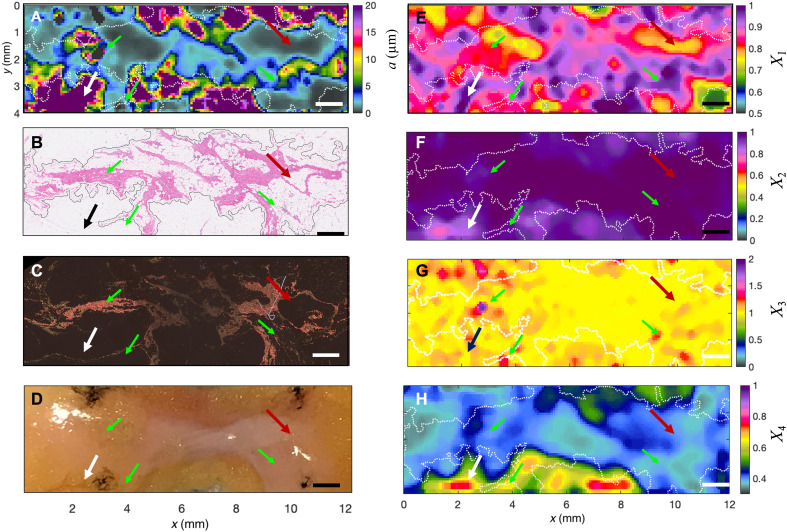
Particle size mapping in benign fibroadipose breast tissue. (**A**) Spatial map of *a* measured by SPARSE. Regions of increased particle size correspond to fat globules in breast tissue (black or white arrows), whereas areas of reduced size align with fibrous structures (maroon arrows). (**B**) H&E section shows the fibrous (maroon arrow) and adipose (black arrow) regions. (**C**) PSR section shows corresponding collagen-rich regions. (**D**) Photograph displaying the gross pathology of fibroadipose tissue. (**E**) Spatial map of *X*_1_ showing that this metric is slightly lower in the regions that correspond with fibrous tissue (maroon arrow) compared to adipose regions (white). (**F**) Spatial map of *X*_2_, exhibiting slightly increased values in fibrous areas. (**G**) Spatial map of *X*_3_, displaying values that are close to 1 across the tissue except for isolated specks of higher values that occasionally coincide with fibrous structures (green arrows), likely due to substantial thinning of collagen fibers in these areas. (**H**) Spatial map of *X*_4_, presenting the reduction of this metric in the fibrous regions compared to surrounding adipose tissue. Dashed lines display the contours of fibrous regions. Scale bars, 1 mm.

**Fig. 7.**
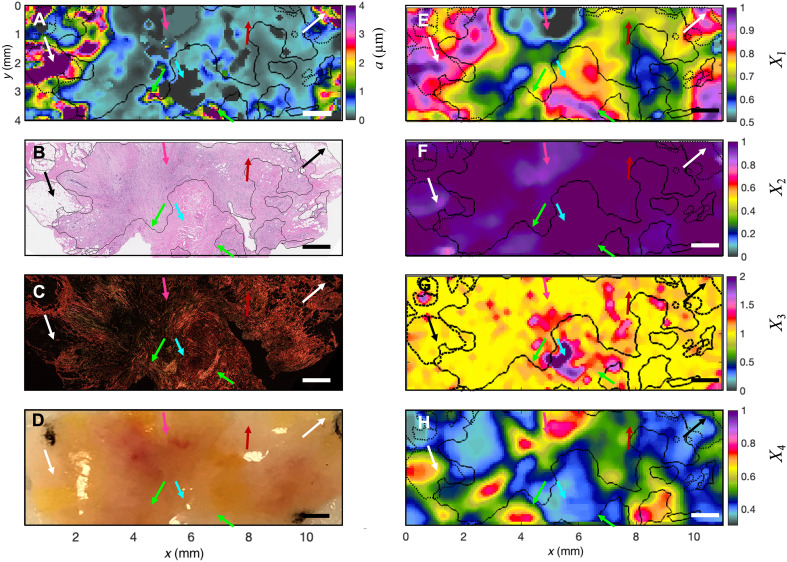
Particle size distribution in an invasive breast carcinoma microenvironment. (**A**) Spatial map of particle size, *a*, measured by SPARSE. Larger sizes correspond to fat globules in the periphery (black and white arrows). The cyan to dark blue hues, (200 nm to 1 μm, maroon arrow) mark stromal fiber–rich regions. Size drops to 100- to 200-nm range in tumor-rich zones (gray hues, magenta arrow), and to <100 nm in isolated islands (cyan arrow), surrounded by bands of *a* > 4 μm (green arrows). (**B**) Matching H&E section shows infiltrating tumor cells interspersed with stromal fibers. A necrotic, scarred center (cyan arrows) is evident. Tumor invasion into fibroadipose structures occurs in the periphery (black arrows). Tumor-to-stroma ratio varies; maroon arrows indicate collagen-rich regions, magenta arrow points to cellular areas. (**C**) PSR section reveals think, bundled collagen (bright orange/red, green arrows) contrasting with thin denatured green elastic debris, generated by tumor cells (cyan arrows). Variations in tumor-to-stroma ratio are noted. (**D**) Photograph shows gross pathology of a breast carcinoma specimens. Tumor vasculatures appear red (magenta arrow). (**E**) Spatial map of *X*_1_ shows reduced values within invasive areas, containing tumor cells and denatured collagen, lacking adipocytes and thick fibers. (**F**) Spatial map of *X*_2_ displays lower values in highly cellular areas. (**G**) *X*_3_ is nearly 1 throughout, except in hollow PSR region (cyan arrow). (**H**) Spatial map of *X*_4_ shows reduced values in fibrous regions. Solid lines mark epithelium contours; dashed lines indicate adipose regions. Scale bars, are 1 mm.

Our results in polystyrene microbeads, milk, and whole blood above demonstrate the capability of SPARSE in evaluating the average particle size in homogenous fluid suspensions that exhibit a range of *n_rel_* and particle concentrations and, in turn, optical properties, including substantial absorption. Next, we test the capability of the SPARSE approach for particle sizing in heterogenous breast tissue specimens by scanning the laser beam across the sample and measuring local polarization attributes of speckle patterns to ultimately reveal spatial maps of particle radii, *a*, distributions (Figs. 6 and 7).

Figure 6 displays the SPARSE size map (Fig. 6A), the corresponding hematoxylin and eosin (H&E) (Fig. 6B) and picrosirius-red (PSR)–stained section (Fig. 6C), and the gross pathology (Fig. 6D) of benign fibroadipose breast tissue. Histology of normal fibroadipose breast tissue typically involves a network of wavy collagen fibrils with radii ranging from 25 to 250 nm, interspersed with large adipocytes of approximately 20-μm radius (*56*, *57*). The H&E slide of Fig. 6B reveals this arrangement of fibrous and adipose compartments, whereas the PSR section of Fig. 6C highlights the presence of thin, undulating collagen fibrils within a single section (maroon arrows). A contour of fibrous region is calculated from the H&E image and overlaid on the SPARSE map of Fig. 6A to facilitate following the histological feature. Visual inspection uncovers smaller structures below 1 μm in fibrous areas (maroon arrows), in notable contrast with larger structures exceeding 20 μm, coinciding with adipose regions (black or white arrows). This wide range of particle sizes encountered in normal fibroadipose tissue are better appreciated from the histogram of size distribution and a zoomed-in view of the H&E sections (fig. S10). Distinguished regions identified within the spatial maps of *X*_1_ to *X*_4_ in Fig. 6 (E to H) correspond to various tissue compartments. Notably, areas with diminished *X*_1_ align with fibrous tissue in both the H&E and PSR sections, likely due to smaller size of collagen fibrils in these areas. Conversely, elevated *X*_2_ coincides with fibrous areas in the gross pathology photo. In contrast, *X*_3_ remains nearly constant across the tissue, except for sporadic elevated specks that occasionally align with fibrous structures, and likely match the thinnest fibrillar structures (cyan arrows). Last, regions of reduced *X*_4_ correspond to the fibrous compartment because smaller sizes are frequently associated with reduced *X*_4_. The individual *X*_1_ to *X*_4_ capture only isolated features of the histology and thus are not expected to present a one-to-one correlation with tissue ultrastructure.

However, it is intriguing that the size map (Fig. 6A) arising through identifying the local cluster and combining these metrics via the corresponding particle size estimation, yields an exquisite map of *a* variations that closely mirror the histopathological features. In particular, the gray to blue regions in the size map highlight submicrometer structures that correlate closely with the whereabouts of the collagenous component. On the other hand, the dark purple areas in the size map coincide with the adipose regions, suggesting that *a* ≥ 20 μm in these areas. However, because of the finite beam spot size, scanning pitch, and the volume-integrated nature of the SPARSE measurement, in contrast to the single 7-μm-thick histopathological sections, small microstructural differences are observed.

Next, SPARSE is further evaluated in an invasive ductal carcinoma specimen obtained from a patient with breast cancer (Fig. 7). Contours of epithelia and adipose regions are calculated from the H&E image and overlaid on the SPARSE map. The color bar range is reduced to 0 to 4 μm to better visualize the smaller size scales emerging in the carcinoma compared to normal specimen. The presence of these markedly smaller sizes is likely due to the abundance of tumor cells and their subcellular structures, as well as the breakdown of collagen meshwork to ultrathin fibrils (fig. S10). The SPARSE map of Fig. 7A features a region of larger particle size in the peripheral adipose region (*a* >> 4 μm) as shown by the black and white arrows. Also featured in the map are cyan to navy blue regions of *a* = 200 nm to 1 μm highlighted by maroon arrows that coincide with intratumoral stroma and match the pink collagen hues in the H&E section (Fig. 7B) and brighter red hues in the PSR image (Fig. 7C). SPARSE further reveals a region embedded in the tumor core that exhibits an abrupt size drop to below 100 nm, denoted by the cyan arrow. Comparison with the H&E image of Fig. 7B reveals the presence of the necrotic area devoid of cells that over time had transformed into scar tissue composed of fissured collagen network. This region of markedly reduced size is encircled by a few isolated islands of larger structures up to and exceeding 4 μm. Examining the corresponding area within the PSR section reveals bundled thick collagen fibers surrounding the necrosed region, as highlighted by green arrows. Another conspicuous compartment, identified in the SPARSE map, features 100 nm < *a* < 200 nm, as highlighted by the magenta arrow.

The matching H&E section reveals the intermediate-grade tumor cells in this region, which invade in a trabecular pattern into the peripheral adipose tissue. Therefore, one would expect that larger size of tumor cell nuclei in this region, 1.5 to 2 times of an RBC in the order of 6 μm, to dominate the size in this region (*58*, *59*). However, close examination of corresponding H&E image and the gross photo suggest the presence of the tumor epithelium, intratumoral stromal fibers, and vasculature in this region. Hence, speckle is formed not only by the large nuclei of tumor cells and the RBCs but also the cell membranes and organelles as well as denatured collagen fibrils. Therefore, the considerable heterogeneity and polydispersity of the breast tissue causes the SPARSE map to reflect the average particle size of the neighborhood. As in Fig. 6 above, the spatial maps of *X*_1_, *X*_2_, *X*_3_, and *X*_4_ overall show variations with the tissue histopathology. For instance, *X*_1_ is higher in fibrous and adipose regions compared to tumor epithelium. This is particularly accentuated in the region pointed to by the magenta arrow. Reviewing all four metrics in this region suggests that the relative values of *X*_1_ to *X*_4_ here (*X*_1_ ~ 0.6, low; *X*_2_ = 0.85, low; *X3* ~ 1; *X*_4_ = 0.85 high) somewhat resembles the trends seen in the RBCs of whole blood above, in agreement with the presence of blood in gross pathology. However, the smaller scattering particles muddle the *X*_1_ to *X*_4_, causing them to deviate from what is expected from larger RBCs or tumor cell nuclei. Conversely, in the stromal components, identified through the PSR image, *X*_1_ lies in 0.7 to 0.95 range, *X*_2_ ~ 1, *X*_3_ varies between 1 and 2, and *X*_4_ = 0.5. This reveals considerable heterogeneity in the particle size, consistent with the thick collagen fiber bundles interspersed with fissured and decomposed fibrils within the tumor stroma. In particular, specks of *X*_3_* ~ 2* coincide with foci of collagen breakdown, whereas regions of maximized *X*_1_ match the thicker bundled collagen strands. Therefore, as seen in Fig. 6, *X*_1_ to *X*_4_, while each capturing certain isolated aspects of tissue ultrastructure, represent only a coarse correlation with tissue morphology. Intriguingly, however, when these metrics are used to identify the local cluster and are substituted in the corresponding particle size estimation equation, the spatial distribution of *a* emerges, which varies in an exquisite harmony with the histopathological landscape of the tumor tissue specimen.

## DISCUSSION

Particle size characterization over a wide range of scales is a universal unmet need across various scientific disciplines, including pharmaceuticals, biotechnology, and clinical medicine. Conventional techniques like DLS and LD are typically limited to low-viscosity, dilute samples, offering minimal utility in biomaterials due to the complexity involved in detecting high-resolution angular scattering distributions or capturing light dynamics over extended time frames (*5*, *13*). To ameliorate these deficits of traditional techniques, alternative approaches have been recently investigated to permit measuring the particle size within inorganic turbid specimens. For instance, a low-cost miniaturized device was developed as an inexpensive alternative to LD in concentrated specimens on the basis of a spatial array of apertures with assorted diameters, each admitting distinct sets of scattering angles (*60*). A machine learning model corrected for multiple scattering and predicted the size (*60*). However, demonstrations were limited to measuring the median diameter of monodispersed large glass beads in the 13- to 125-μm range, while also requiring prior knowledge of bead concentration and, as a result, not conducive for biological specimens with smaller size scales and unknown particle concentrations or optical properties (*60*). More recently, other groups demonstrated that the spatial autocorrelation of speckle informed the particle size distribution of powdered solid drugs in the 100- to 500-μm range (*21*). Nevertheless, this feature of laser speckle was most informative for sizing powdered drugs of larger grain sizes spread on a surface during processes such as drying, blending, and milling and presented limited applicability to biomaterials (*21*). In parallel, translational technologies have been proposed on the basis of identifying the unique signatures of the scattered light, such as resonance spectra or angular distribution, which inform the particle size in native, intact tissues and biomaterials (*18*, *61*, *62*). For instance, a/LCI exploits coherence gating and sums the intensity scattered from each depth section to obtain the corresponding angular profile. This profile in then fitted to precomputed Mie scattering phase functions for 2.5- to 9-μm particles, enabling accurate evaluation of nuclear size and in vivo detection of dysplasia (*18*, *19*). While affording high accuracy in early detection of epithelial neoplasia, this technique remains mostly focused on nuclear ultrastructure (*20*). Moreover, the depth resolution of a/LCI is offset by reduced penetration depth and complexity, and the angular averaging of scattered light compromises lateral resolution (*63*). We and others had previously observed the feasibility to qualitatively estimate particle sizes through comparing the azimuth angle variations of ${\hat{I}}_{\|}$ with a look-up table (LUT) (*29*, *36*). However, this only allowed for semiquantitative, discrete size estimation within the 250 nm–to–2.5 μm range, primarily distinguishing sizes slightly smaller or larger than the wavelength based on whether the scattering follows Rayleigh or Mie formalisms, and was limited to purely scattering samples with generally known refractive indices (*29*, *36*).

Here, we introduce a breakthrough technique, SPARSE, which measures the average particle size and maps the size distributions within intact tissues and biomaterials across a broad range of sizes from 10 nm to 10 μm. Unlike existing tools that strictly isolate single-scattered light and avoid diffusely scattered light at all costs, be it through sample dilution, coherence gating, or advanced learning techniques, SPARSE leverages diffuse multiple scattering as a beneficial asset. By capturing spatial and temporal variations in polarized laser speckle patterns, created by the rich multiple scattering of coherent light from intrinsic particles, SPARSE enables precise sizing across various biological materials. In addition, where conventional methods require complex, costly multiangle detection setups, SPARSE achieves sensitive, noninvasive particle sizing with streamlined instrumentation: It uses commercially available CMOS cameras and simple polarization adjustments to capture wide-field polarized speckle patterns directly from the sample. Spatiotemporal modulations of speckle intensity, captured by the cameras featuring polarizers of orthogonal orientations reveal the distinctive temporal fluctuation rate and spatial intensity distribution at each polarization state, allowing SPARSE to correlate these variations with particle size, concentration, and refractive index without invasive sample preparation.

The temporal attributes of polarized speckle intensity fluctuations in biofluids and tissue arise from thermally driven, microscale displacements of endogenous scatterers. These random, Brownian motions are often quantified by the mean square displacements, $\langle \;∆r^{2}\left(t\right)\;\rangle .$In viscous fluids, diffusive motion of particles yields $\langle \;∆r^{2}\left(t\right)\;\rangle =6Dt$, where *D* is the particles diffusion coefficients. This provokes monotonically decaying *g*_2⊥_(*t*) and *g*_2||_(*t*). Conversely, in viscoelastic soft tissue, the particles’ subdiffusive motion results in a power law behavior, $\langle \;∆r^{2}\left(t\right)\;\rangle =t^{\alpha \left(t\right)}$, and induce multiple relaxations in g_2⊥_(*t*) and g_2||_(*t*) curves. In more elastic or cross-linked environments, thermal motions reduce to smaller vibration, i.e., $\langle \;∆r^{2}\left(t\right)\;\rangle =r_{0}^{2}$, considerably slowing down the *g*_2⊥_(*t*) and *g*_2||_(*t*) decorrelations. SPARSE quantifies the temporal attributes, using the metric $X_{3}=\frac{\text{log}\left[g_{2⊥}\left(t\right)\right]}{\text{log}\left[g_{2∥}\left(t\right)\right]}$. Hence, the ratio of *g*_2⊥_(*t*) and *g*_2||_(*t*) cancels motion-related terms, leaving only the differences in photon path lengths and, in turn, the particle size. However, both dynamics and size still determine the relevant timescales and frame rates required to compute *X*_3_ accurately. For fast-moving particles in low-viscosity media (e.g., microspheres in water-glycerol or milk), frame rates >7000 frames per second (fps) were required to capture rapid speckle fluctuations, with each frame capturing only a single, fully developed, high-contrast speckle realization. This ensured that *g*_2_(*t*) is temporally resolved and spanned the full decorrelation range. In contrast, for slowly decorrelating speckle from viscoelastic breast tissue, lower frame rates (e.g., 486 fps) yield sufficient temporal sampling of the speckle fluctuations. Abundance of multiple scattering in SPARSE permits extremely small displacements in the order of 1 to 10 nm, generated by thermal energy in stiffer tissues with elastic moduli, of up to at least ~10 to 100 kPa to remain resolvable and elicit measurable speckle dynamics needed for calculating the *X*_3_ (*32*). This presents and advantage over single scattering techniques like DLS, where considerable Brownian excursions are needed to induce phase shifts and speckle dynamics.

SPARSE estimates particle size, without requiring prior knowledge of refractive index, using the size estimation equations created from the synthetic library of quantifiable metrics. These key metrics were derived from Monte Carlo simulations of polarized speckle patterns for their strong and complementary sensitivity to size. Unlike prior approaches that relied on a single feature to approximate either particle size or optical properties from the diffusely scattered light, these metrics collectively capture both spatial and dynamic information, enabling accurate size estimation across a broad range (10 nm to 10 μm) while minimizing confounding effects of optical properties.

Additional features of polarized speckle that capture subtler size variations to further enhance SPARSE may exist. One key speckle attribute, identified in this work but not explicitly quantified, is the radial location of peak intensity of ${\hat{I}}_{⊥}$, an indicator of *n_rel_*. While this attribute does not directly report particle size, it helps SPARSE infer the appropriate size estimation model from its library, effectively adapting the algorithm to variations in *n*_rel_ without requiring it as an external input.

Because the relationships between *X*_1_–*X*_4_ and particle size are non-linear and vary across the optical properties and size range, no single global equation could adequately describe the mapping from these metrics to the particle size. Cluster analysis was therefore used to partition the synthetic *X*_1_–*X*_4_ space into five distinct regions, where local trends were more consistent, enabling a step-wise regression to derive a separate size estimation equation tailored to that subset of data. This strategy improved estimation accuracy and ensured robustness across the broad range of optical properties and size scales. To support the particle size prediction models of SPARSE, multiple synthetic libraries of *X*_1_–*X*_4_ were developed assuming spherical particles, exhibiting various *n_rel_* (e.g., 1.034, 1.067, and 1.1) with respect to their microenvironment. Monte Carlo simulations confirmed that the *n_rel_*-dependent shift appears prominently in ${\hat{I}}_{⊥}$, which SPARSE implicitly uses through its model selection approach to infer particle size. These *n_rel_* values are within the limited range of *n_rel_* = 1.0 to 1.2 that arise in biological specimens due to their high-water content (*37*, *40*, *41*).

To demonstrate the capacity of SPARSE to operate across this *n_rel_* range without relying on prior knowledge of refractive index values, we designed experiments using polystyrene microspheres suspended in aqueous glycerol mixtures with controlled refractive indices, producing *n_rel_* ≈ 1.09 to 1.12. Together with lipid globules (*n_lipid_* = 1.46, *n_medium_* = 1.33, and *n_rel_* ≈ 1.10) and casein micelles (*n_casein_* ≈ 1.57, *n_medium_* = 1.33, and *n_rel_* ≈ 1.18) within the milk specimens, these specimens covered the *n_rel_* = 1.1 to 1.18 range (*45*, *46*). Similarly, the literature reports on the refractive indices of adipose and glandular breast tissue (*n_adipose_* = 1.455 and *n_glandular_* = 1.4), together with our demonstrations of the consistency of SPARSE measurements with histological features in human breast tissue specimens further confirmed the validity of SPARSE measurements in the *n_rel_* ≈ 1.09 to 1.19 range. The lower range of *n_rel_* < 1.09, encountered in biological specimens, were covered by the blood specimens of modified tonicity. Specifically, the whole blood served as a natural *n_rel_* = 1.03 case, with RBCs (*n* ≈ 1.402) suspended in plasma (*n* ≈ 1.36). Moreover, the osmotic shifts in blood samples, modulated *n_rel_* in 1.03 to 1.1 enabling a dynamic validation in this range. Together, the convergence of SPARSE results with those from DLS, smear microscopy, and histology across all sample types and *n_rel_* values supports the conclusion that SPARSE can accurately estimate particle size without requiring explicit input of the sample’s refractive index.

SPARSE measurements in standard polystyrene microspheres exhibited strong, statistically significant correlation with DLS. Deviations at *a <* 100 nm occurred likely because the particle size estimation equation at this range is primarily driven by *X*_3_. As explained earlier, all the metrics, including *X*_3_, depend on *a*, μ*_a_*, and μ*_s_*′. Yet, at smaller sizes, μ*_s_*′ itself drastically varies by *a*. More specifically, for a given particle concentration, as *a* drops below 100 nm, the scattering cross section and, in turn, μ*_s_*′ reduce substantially. Therefore, a competing effect emerges between the reduction of *a* and μ*_s_*′ on *X*_3_, reducing the sensitivity of this metric to size variations at these minute scales. Consequently, a large *X*_3_ may be equivocally interpreted as a moderate concentration of minute particles or a dense concentration of slightly larger scatterers, causing the estimated size to deviate from DLS measurements.

SPARSE was successful in characterizing the average size of spheroidal fat globules and protein micelles in milk samples. As mentioned above, while the fat globules indeed elicited an *n_rel_* = 1.1, protein micelles were more refractive exhibiting *n_rel_* = 1.18. However, no shift in the radial intensity peak of the was detected in ${\hat{I}}_{⊥}$ of our milk measurements, indicating that switching to a different particle size estimation model based on *n_rel_* variation is not needed. This observation is further supported by MCRT simulations, which show that for particle sizes below 500 nm, the *X*_1_–*X*_4_ metrics are largely invariant to modest changes in *n_rel_* (Supplementary Materials). Consequently, the size estimation equations derived for *n_rel_* ≈ 1.1 remains valid in milk, where the dominant scatterers fall within this sub–500 nm range. Moreover, homogenization reduces size variability among fat globules, minimizing polydispersity and enhancing the applicability of a single SPARSE model. In addition, the ability of SPARSE to detect the few nanometer shifts in size caused by increasing the fat concentration from 0 to 4% demonstrated the acute sensitivity of this approach in the 100- to 200-nm range, as validated through comparison with DLS measurements. On the other hand, DLS declared an average size that significantly correlated with SPARSE but also revealed the distinct sizes of protein and fat particle populations. Nevertheless, to conduct DLS measurements, samples were extensively diluted in deionized water, whereas SPARSE measured the size in untampered milk phantoms, suggesting the utility of this approach for monitoring the texture of food products composed of spheroidal particles of unknown concentrations.

Compared to polystyrene microspheres and milk, sizing RBCs in whole blood posed additional challenges due to changes in both real and imaginary parts of *n_rel_* and, in turn, μ*_a_* and μ*_s_*′, concurrent to *a*. The challenge was readily apparent in isotonic blood of 0.9% NaCl concentration, where the elliptical envelope of ${\hat{I}}_{\|}$ resulted in *X*_1_ = 0.66, which in isolation suggests a small *a*. However, by collectively considering this attribute in the context of other metrics (*X*_2_–*X*_4_), SPARSE successfully yields *a* = 2.2 μm, which closely agrees with smear microscopy measurement of *a* = 2.29 μm and the reported value of 2.3 μm in swine (*54*). The lower *n_rel_ =* 1.03 in isotonic and mildly hypertonic swine blood, i.e., NaCl concentrations of 0.9 and 1.08%, caused the scattering events to be increasingly forwardly directed and the optical paths of backscattered rays to be considerably longer compared to polystyrene microspheres and milk samples above. This in turn enhanced the impact of absorption in terminating the longer optical paths, to an extent that was not recapitulated in the synthetic data based on MCRT assuming *n_rel_ =* 1.1. Nevertheless, the reduced *n_rel_* of swine blood, spiked with 0.9 and 1.08% saline solutions, was readily apparent from ${\hat{I}}_{⊥},$ given the shifted the maximum intensity from the envelope centroid to the middle of the leaflets. This prompted the SPARSE to accommodate for the lower *n_rel_* by switching to the corresponding particle size estimation equations, previously developed for the reduced *n_rel_* = 1.03. As saline concentration increased above 1.25%, μ*_a_* was reduced because of conformational changes in heme groups, while *n_rel_* between RBCs and plasma simultaneously increased. This in turn contributed to an overall rise in the μ*_s_*′. The enhanced *n_rel_* yield the synthetic library and the particle size estimation equations, derived on the basis of *n_rel_* = 1.1, applicable to the experimentally measured metrics and trace the reduction in RBC size at higher NaCl concentration, in close agreement with smear microscopy measurements. While blood samples were considerably polydisperse, RBCs accounted for much of the speckle signal. However, RBCs are not spherical and instead typically have a discoid shape. Yet, SPARSE was able to track the shift in the sphere-equivalent radii of RBCs in whole blood in its native state, likely because rich multiple scattering from numerous randomly oriented RBCs inherently averaged out the contribution of short and long dimensions and shape features on the scattering signal. In contrast, DLS did not fare well for quantifying cell size distributions in whole blood, which is a complex, light-absorbing polydisperse medium containing irregularly shaped, nonspherical cells. For this reason, SPARSE measurements in blood were benchmarked against RBC size distributions derived from stained blood smear microscopy as a traditional reference. A strong, statistically significant correlation was observed between SPARSE and smear microscopy size measurements (*R* = 0.73, *P* < 0.007). However, smear microscopy was less sensitive to size changes, showing little reduction between 1.25 and 1.4%, and between 1.75 and 2.1% NaCl concentrations, likely due to its two-dimensional (2D) nature. In addition, swine blood, being less resilient to osmotic shock than human blood, presented signs of hemolysis under increasingly hypertonic conditions. SPARSE likely interpreted ghost cells and membrane remnants as smaller scattering particles, whereas smear microscopy captured them as pink smudges. Despite aggressive thresholding, some of these artefacts remained, leading to RBC size overestimation. Consequently, shrinkage appeared more pronounced in SPARSE.

Following demonstrations in microspheres, milk, and blood, we investigated the potential of SPARSE to enable real-time, label-free assessment of structural size distributions in unprocessed tissue, ultimately aiming to address key limitations in standard diagnostic evaluation of epithelial neoplasm. The central challenge in this space is that visual assessment of nuclear pleomorphism in H&E-stained slides remains poorly reproducible because of interobserver variability (*64*). Efforts to improve consistency using digital pathology and AI are further challenged by artefacts introduced during fixation, sectioning, and staining (*65*, *66*). Moreover, the current diagnostic workflow does not yet incorporate the ECM architectural features, such as those defined by tumor-associated collagen signatures, despite their known prognostic value (*67*). Unlike the relatively homogeneous biofluids previously assessed with SPARSE, breast tissue specimens presented a more complex context given the variations in size, *n_rel_*, absorption, and particle concentration across the specimen. Nevertheless, tightly focusing the beam and scanning it across the sample minimized the influence of these variabilities within the illumination volume, causing the model to remain applicable at each scanning point. Furthermore, rich multiple scattering in tissue implied that the emergent speckle could be treated as the superposition of speckle patterns from different monodisperse populations, causing the SPARSE prediction model developed using a synthetic library of monodispersed homogenous turbid medium to remain resilient to polydispersity. This is evidenced by our results in normal fibroadipose tissue, which elucidate the characteristic size distribution of adipocytes and collagen fibrils, in their corresponding compartments identified in the H&E and PSR images. Compared to normal fibroadipose tissue, invasive ductal carcinoma presented a greater deal of size heterogeneity, as evidenced by histological analysis, which could likely complicate the size estimation. However, SPARSE size maps exhibited a detailed correspondence with histopathological features and varied in harmony with tissue compartments of varying morphologies. Figure 7A demonstrates that the relative size differences nicely isolate histologically distinct regions in the highly heterogeneous breast carcinoma specimen. Unlike fibroadipose tissue, which showed size distributions skewed toward larger particles due to the presence of 10 to 150 μm of adipocytes (*68*), tumor tissue exhibited a peak at smaller sizes (0 to 5 μm), reflecting densely packed epithelial cells with high nuclear-to-cytoplasmic ratios and organelle-rich cytoplasm (fig. S10). Nevertheless, accurate absolute values were in some cases harder to estimate. For instance, the difficulty of distinguishing cell nuclei size is likely caused by the abundance of other organelles, membranes, and collagen fibrils, overshadowing the signal emanated from the nuclei within the illuminated volume. In the future, using a smaller beam spot and smaller scanning steps could likely increase the resolution and contrast of SPARSE measurements to permit isolating absolute values of different size populations.

The current study has demonstrated SPARSE as an innovative particle sizing tool with a range of valuable applications, yet some limitations warrant consideration. First, SPARSE’s sensitivity to size variations is reduced at the extremes of the measurable size range, particularly below 100 nm and above a few micrometers, as seen in deviations within the scatter diagram of Fig. 3M and in the necrotic tumor core and adipose regions in breast tissue size maps of Figs. 6A and 7A. This limitation arises because of reduced sensitivity of the key metrics, e.g., *X*_1_ and *X*_3_, within clusters 1, 4, and 5, corresponding to the lower and upper bounds of the SPARSE measurement range. SPARSE’s size range could be expanded through further technical enhancements by using tunable wavelength source to varying the a/λ ratio, and in turn broaden the range of particles sizes that could be accessed (*5*).

Second, SPARSE’s lateral mapping through beam scanning provides precise size distribution in heterogeneous tissues. However, this approach may not be conducive for resolving the full extent of size variations in highly polydisperse biofluids or exceedingly heterogeneous soft tissue, where the scales of heterogeneities are smaller than the mean free path of light diffusion, causing the polydispersity within the illuminated volume to substantially modulate the *X*_1_–*X*_4_. In this respect, considering speckle patterns as a simple superposition of signal due to subpopulations of small and large particles, ${\hat{I}}_{\|}$ is expected to be influenced primarily by smaller particles that contribute shorter optical paths due to their isotropic scattering. As a result, *X*_1_ likely represents the smaller size populations, approximately within the *a ≤* λ size range. This may be accentuated in biological tissue, where the scattering signal from numerous smaller particles overshadows the few larger scattering centers. Conversely, ${\hat{I}}_{⊥}\;$may be dominated by larger forwardly scattering particles in *a >* λ–nm range, for which the longer optical paths cater to cross-polarized light. Thus, *X*_2_ and *X*_4_ could be biased toward larger particles in the population. Considering differences in the contributions of size scales to co- and cross-polarized speckle, *X*_3_ could present a different behavior in polydisperse and heterogeneous specimens. This is because the copolarized speckle is contributed by shorter paths involving smaller particles. On the other hand, cross-polarized speckle is likely contributed by longer paths encountering larger particles. In addition, decay rates of *g*_2||_(*t*), and *g*_2⊥_(*t*) at early and long times are modulated by faster and slower dynamics, elicited by smaller and larger particles, encountered in longer and shorter paths, respectively. Together, these suggest a time-variant log[g_2⊥_(*t*)]/log[g_2||_(*t*)], causing *X*_3_ to take on a wider range of values due to additional implications imparted by polydispersity. These insights need to be considered when priming the SPARSE for applications in highly polydisperse biological specimens where multiple size populations coexist within a minute sample volume on the scales of a fraction of one cubic millimeter (~0.001 mm^3^).

Last, SPARSE’s measurements are resilient to refractive index variations at smaller sizes (<500 nm), where scattering is mostly isotropic and variations in *n_rel_* does not considerably modify the scattering phase function, and consequently the ${\hat{I}}_{\|}$ and ${\hat{I}}_{⊥}$ (fig. S1). However, as particle size increases, reduced *n_rel_* values can result in more forward-directed scattering, affecting angular distributions and altering ${\hat{I}}_{\|}$ and ${\hat{I}}_{⊥}$ and in turn *X*_1_, *X*_2_, and *X*_4_ intensity metrics (*69*, *70*). Here, we identified the signature of reduced *n_rel_* as the ${\hat{I}}_{⊥}$maximum occurring at the center of lobes away from its centroid, which enabled us to modify the particle size estimation equation accordingly. Identifying these shifts could guide future iterations of SPARSE, with additional metrics to account for variations in refractive index, ensuring robust performance across a wider range of biological contexts.

In summary, SPARSE offers an innovative platform for particle sizing across multiple disciplines, supporting both current needs and offering potential for future improvements that could further enhance its applicability. SPARSE’s unique approach leverages multiple scattering—a feature typically avoided in conventional techniques—to capture information over a broad particle size range (10 nm to 10 μm) directly within tissues and biomaterials. This transforms a traditionally challenging aspect of rich light scattering into an asset, offering wide-field speckle patterns that reveal detailed, intrinsic nano- and microstructural properties across diverse specimens. Together, SPARSE’s wide-ranging applicability across fields supports critical advancements in science, health care, and industry, that may likely establish it as a uniquely versatile and impactful tool for particle sizing in complex biological and material systems.

## MATERIALS AND METHODS

### Experimental design

The design, development, and validation of the SPARSE approach for the size characterization of tissues and biomaterials involved several essential steps. We first designed and built the SPARSE optical instrument to illuminate specimens and capture back-scattered speckle frame series in both parallel and perpendicular polarization states relative to the polarization axis of the illumination beam, to provide a comprehensive view of the spatiotemporal variations in polarized speckle patterns. Alongside hardware development, we conducted polarized light correlation-transfer MCRT (PLCT-MCRT) simulations to model the diffused intensity envelope of polarized speckle, i.e., ${\hat{I}}_{\|}$ and ${\hat{I}}_{⊥}$ and its temporal autocorrelation curves, *g*_2||_(*t*) and *g*_2⊥_(*t*) in turbid media of varying optical properties (μ_s_*′*, 0.25 to 4 mm^−1^; μ*_a_*, 0 to 70% μ_s_′) and scattering particle size (*a*: 10 nm to 10 μm). Using the simulated spatiotemporal attributes, we calculated a synthetic library of metrics [*X*_1_, *X*_2_, *X*_3_, *X*_4_] for the full range of parameters [*a*, μ*_a_*, μ*_s_′*]. The synthetic metrics were then clustered, and for each cluster, we derived an equation to estimate the particle size from the metrics [*X*_1_, *X*_2_, *X*_3_, *X*_4_]. To evaluate the particle size within biological specimens, we simply followed the workflow illustrated in Fig. 1. The specimen was evaluated using the SPARSE instrument to experimentally acquire the speckle frame series and characterize the spatiotemporal attributes of the polarized laser speckle, i.e. ${\hat{I}}_{\|}$, ${\hat{I}}_{⊥}$, *g*_2||_(*t*), and *g*_2⊥_(*t*). From these attributes, the experimental metrics ([*X*_1_, *X*_2_, *X*_3_, *X*_4_]) were calculated and the appropriate cluster for the sample was identified. The metrics were then substituted into the equation corresponding to the identified cluster to estimate the particle size. To validate the SPARSE, biofluid and tissue specimens were prepared under controlled and reproducible conditions. Particle size values evaluated by SPARSE were compared with those obtained using DLS for polystyrene beads and milk phantoms, smear microscopy for swine blood specimens, and histopathological features for solid soft tissues. The experimental protocols and technical details for each step are detailed below and further expanded in the Supplementary Materials.

### SPARSE optical setup

Figure 1A displays the schematic diagram of the SPARSE optical setup. A polarized laser beam (Thorlabs, HNL210LB, 633 nm, 21 mW) was collimated and expanded to a beam diameter of 1 cm, passed through a focusing lens (Thorlabs, LA1986-A, Plano-Convex, AR coated, *f* = 124.6 mm), a beam splitter (Thorlabs, BS013, nonpolarizing, beam splitter cube), resulting in a 10-μm spot of 5 mW on the sample surface. Backscattered light from the sample was collected using a macro lens (Computar, MLH-10X) and imaged onto the sensor of a high-speed CMOS camera (Basler, acA2000-340 km, Germany). A rotatable linear polarizer placed in front of the macro lens enabled sequential acquisition of co- and cross-polarized speckle patterns by adjusting the detection polarization angle. The Camera Link interface of the primary camera allowed rapid frame acquisition, ensuring capture of fully developed, high-contrast speckle patterns generated from dynamic fluid samples. To measure speckle patterns at orthogonal polarization states, the detection polarizer was rotated by 90°, and the measurements were repeated. For soft tissue imaging, the setup was modified to enable simultaneous acquisition of both polarization channels by incorporating two additional CMOS cameras (Basler, acA1920-155um, USB 3.0 interface, Germany) with similar sensor characteristics. In this configuration, backscattered light was split into two paths using a beam splitter, and each polarization channel was directed to a separate camera for parallel detection. Adjusting the macro-lens f/# to 11 and magnification to ~1× resulted in a speckle grain size of at least S = 2.44λ(1 + M)f/# = 34 μm and a 1D pixel-to-speckle ratio of 5.7, ensuring sufficient spatial sampling, beyond Nyquist theorem (*71*). The acquisition frame rate and exposure time were selected according to the dynamics of the evaluated sample to ensure sufficient temporal sampling and adequate speckle contrast. Specifically, for milk specimens, the frame rate was set to 7142 fps (exposure time = 103 μs). On the other hand, for whole blood specimens, the frame rate was set at 3300 fps (exposure time = 266 μs). For polystyrene microsphere suspensions in aqueous glycerol mixtures, a minimum frame rate of 1808 (exposure time = 513 μs) was used. To permit spatial mapping in heterogeneous biological tissues, the sample was placed in a holder and mounted on a two-axis motorized stage (Newport, MFA-CC), controlled by a motion controller (Newport, ESP301). The sample was scanned in a serpentine pattern, with a scan pitch of 150 μm, to cover an area of a few square millimeters. At each scanned spot, speckle frames were acquired at 486 fps (exposure time = 2000 μs) for a sensor region of interest (ROI) of 1.2 mm by 1.2 mm, for 2 s. A custom-build C++ acquisition software enabled adjusting the frame rate, exposure time, acquisition time, and frame size and synchronized the motion controller and the cameras.

### SPARSE clustering analysis and particle size estimation

We exploited our previously developed PLCT-MCRT approach to simulate the propagation of a focused laser beam in a turbid media exhibiting *a*: 10 nm to 10 μm, μ*_s_′*: 0.5 to 4 mm^−1^, and μ*_a_*: 0 to 70% μ_s_′ (Supplementary Methods) (*27*–*29*). From the photon trajectories, the first- and second-order statistics, i.e., ${\hat{I}}_{\|}$, ${\hat{I}}_{⊥}$, *g*_2||_(*t*), and *g*_2⊥_(*t*), of remitted speckle patterns and the corresponding size-dependent [*X*_1_, *X*_2_, *X*_3_, *X*_4_] metrics were simulated (Supplementary Methods). To optimize particle size estimation, we used *K*-means clustering to segment the [*X*_1_, *X*_2_, *X*_3_, *X*_4_] metrics space into five distinct regions, each with tailored particle size estimation equations (Supplementary Materials). We chose this clustering method for its efficiency with large datasets, allowing us to minimize within-cluster variance and iteratively refine centroid placement (*72*). We determined the optimal number of clusters using silhouette scoring, which confirmed that five clusters provided the best partitioning (*73*). Each cluster corresponded to a distinct region in the [*a*, μ*_s_′*, μ*_a_/*μ*_s_′*] parameter space, with smaller particles and low and high turbidity levels populating clusters #1 and #5, respectively, and clusters #2, #3, and #4 corresponding to increasingly larger particles and turbidity levels. Stepwise regression was subsequently applied within each cluster to generate cluster-specific particle size estimation equations, using different metrics and their interactions to minimize error. For less turbid media (cluster #1), a linear regression model was sufficient, while the remaining clusters used exponential models. Pinpointing the cluster centroids and derivation of the particle size estimation equations was a one-time process. Once derived, these equations were readily applicable to predicting the scattering particle size from the experimentally evaluated [*X*_1_, *X*_2_, *X*_3_, *X*_4_] metrics, as detailed below.

### Quantifying size-dependent metrics of the polarized speckle patterns

The entirety of the computation was carried out using MATLAB 2022a. Experimentally acquired speckle frames were processed to obtain the intensity envelopes (i.e., diffuse reflectance profiles) in the co- and cross-polarization states, ${\hat{I}}_{\|}\left(x,y\right)$ and ${\hat{I}}_{⊥}\left(x,y\right)$, as follows

$${\hat{I}}_{∥}\left(x,y\right)=\frac{{\left\langle I_{∥}\left(x,y,t\right)\right\rangle }_{t}}{\text{max}\;{\left\langle I_{∥}\left(x,y,t\right)\right\rangle }_{t}},{\hat{I}}_{⊥}\left(x,y\right)=\frac{{\left\langle I_{⊥}\left(x,y,t\right)\right\rangle }_{t}}{\text{max}\;{\left\langle I_{⊥}\left(x,y,t\right)\right\rangle }_{t}}$$ (1)

where $I_{\|}\left(x,y,t\right)$ and $I_{⊥}\left(x,y,t\right)$ refer to speckle intensities at location (*x*, *y*) and time instance *t*, and ⟨⟩*_t_* denotes temporal averaging. In addition, speckle intensity autocorrelation curves for both co- and cross-polarizations are calculated according to

$$\begin{array}{c} \begin{array}{c} g_{2∥}\left(t\right)=\frac{g_{2∥,\mathrm{raw}}\left(t\right)-1}{g_{2∥,\mathrm{raw}}\left(t=t_{0}\right)-1},\;\\ g_{2∥,\mathrm{raw}}\left(t\right)={\langle \frac{{\langle I_{∥}\left(x,y,t+t_{0}\right)I_{∥}\left(x,y,t_{0}\right)\rangle }_{t}}{{\langle I_{∥}\left(x,y,t+t_{0}\right)\rangle }_{t}{\langle I_{∥}\left(x,y,t_{0}\right)\rangle }_{t}}\rangle }_{x,y}\\ g_{2⊥}\left(t\right)=\frac{g_{2⊥,\mathrm{raw}}\left(t\right)-1}{g_{2⊥,\mathrm{raw}}\left(t=t_{0}\right)-1},\;\\ g_{2⊥\mathrm{raw}}\left(t\right)={\langle \frac{{\langle I_{⊥}\left(x,y,t+t_{0}\right)I_{⊥}\left(x,y,t_{0}\right)\rangle }_{t}}{{\langle I_{⊥}\left(x,y,t+t_{0}\right)\rangle }_{t}{\langle I_{⊥}\left(x,y,t_{0}\right)\rangle }_{t}}\rangle }_{x,y} \end{array} \end{array}$$ (2)

where, raw g_2_(*t*) curves were normalized to the speckle contrast (*74*).

To calculate *X_1_*, ${\hat{I}}_{∥}\left(x,y\right)$ was contoured at 30% of its maximum and the inner and outer circles of the contour were identified. Radial averaging of ${\hat{I}}_{∥}\left(x,y\right)$ in the annular region between the two circles yields ${\hat{I}}_{∥}\left(\varphi \right)$, from which

$$X_{1}=\frac{{\hat{I}}_{\|@\varphi =0{}^{\circ}}}{{\hat{I}}_{@\varphi =90{}^{\circ}}}\;$$ (3)

where φ stands for azimuth angle, and φ=90° corresponds to the direction aligned with the polarization axis of the illumination beam. Likewise, ${\hat{I}}_{⊥}\left(x,y\right)$ was contoured at 30% of its maximum, and the area and perimeter of the contour, *A* and *P*, were evaluated to calculate

$$X_{2}=\frac{4\pi A}{P^{2}}$$ (4)

In addition, the differential decorrelation rate of co- and cross-polarized speckle frame series was quantified via

$$X_{3}={\left.\frac{\text{log}\left[g_{2⊥}\left(t\right)\right]}{\text{log}\left[g_{2∥}\left(t\right)\right]}\right|}_{t=t_{m}}$$ (5)

where *t*_m_ is the instance in time where the temporal derivative of g_2_(*t*) curves were maximized.

Last, the average radius of the ${\hat{I}}_{⊥}\left(x,y\right)$ contour was calculated as

$$X_{4}=\sqrt{\frac{A}{\pi }}$$ (6)

The experimentally measured [*X*_1_, *X*_2_, *X*_3_, *X*_4_] vector of metrics was assigned to one of five the clusters of simulated metrics, which exhibited the shortest Euclidean distance from its centroid. Subsequently, the experimentally measured [*X*_1_, *X*_2_, *X*_3_, *X*_4_] metrics were substituted in the particle size estimation equation, corresponding to that cluster, to yield the scattering particle size.

### Sample preparation

Inert aqueous polarization microsphere suspensions of 10% weight fraction were obtained from the Bangs Laboratories Inc. (NC, USA), exhibiting nominal radii in the range of 50 nm to 4800 nm (*N* = 14). For microspheres smaller than 500 nm, 100 μm of the stock solution was mixed with 900 μm of glycerol, yielding a final polystyrene microsphere weight fraction of 1% in a 90% aqueous glycerol solution. Theoretical calculations, using Mie theory, suggested that μ_s_′ exhibited minimal variations around 1 mm^−1^, starting at 0.77 mm^−1^ for *a* = 75 nm, reaching a peak of 1.08 mm^−1^ for *a* = 100 to 200 nm, and reducing to 0.81 mm^−1^ at *a* = 400 nm. For microspheres 500 nm and larger, the suspensions entailed 3% microsphere, 70% glycerol, and 27% water. The reduced viscosity of the solvent compensated for the increased particle size and reduced the variability in the speckle fluctuations rate, permitting the same practical frame rate and acquisition duration across the size range. It also helped maintain biologically relevant μ*_s_′* values of 3 mm^−1^ for *a* = 500 nm to 0.63 mm^−1^ for *a* = 5 μm. These μ*_s_′* values were only theoretical and used only in the process of phantom design but were not involved in the SPARSE particle size estimation. In practice, μ*_s_′* likely deviated from these baseline values due to experimental factors such as particle sedimentation or aggregation. Additional sets of microsphere phantoms were prepared by suspending microspheres in 60% aqueous glycerol solutions. The microsphere suspensions were placed in Eppendorf tubes and sonicated for 15 min to break down any potential aggregates. Subsequently, the samples are pipetted into custom-made 3D-printed cylindrical wells of 9-mm diameter and 1-cm depth. Speckle frame series were acquired at 3389 (*a* ≤ 500 nm) and 1808 fps (*a* > 500 nm) for 2 s in both parallel and perpendicular polarization states over five different points on the sample surface.

Milk samples were obtained from a local grocery store (Wholefoods Market) at 0% (nonfat), 1% (low-fat), 2% (reduced-fat), and 4% (whole-milk) fat concentrations. The specimens were pipetted into the custom 3D-printed wells for SPARSE measurements.

A 50-ml bottle of EDTA-anticoagulated tube of whole swine blood was purchased (Animal Technologies, USA). A total of five samples were prepared by aliquoting 3 ml of whole blood into 5-ml Eppendorf tubes. The tubes were spiked with 600 μl of saline solutions at concentrations of 0.9, 2, 4, 6, and 8%, yielding final NaCl concentrations of 0.9, 1.1, 1.41, 1.75, and 2.1%, respectively. For each condition, a 1-ml aliquot was set aside for SPARSE measurements. For each condition, three replicates were prepared by placing 60 μl of specimen into custom 3D-printed imaging chambers with a clear polycarbonate window for optical measurement. To prepare blood smears, 2-μl droplets were placed on the glass slide, and a second slide angled at 30° was used to smear the specimen. The slides were air dried for 2 hours and then submitted to the Wellman center Core photo-pathology laboratory, where they were fixed and stained with Modified Wright-Giemsa stain (WG-16, Millipore-Sigma, USA) before being digitized by the Nano-Zoomer whole slide scanner. Under isotonic conditions (0.9% NaCl), swine RBCs retain their characteristic biconcave morphology although smaller than human RBCs. Increasing NaCl concentrations induced hypertonic stress, causing osmotic efflux of intracellular water and resulting in RBC shrinkage ultimately creating crenated morphologies at higher saline concentrations.

Freshly excised deidentified and discarded breast carcinoma and benign normal tissue from matching sites were obtained from the Massachusetts General Hospital (MGH) surgical pathology suite following mastectomy procedures (MGH, IRB#2011P000301). The specimens were stored in phosphate-buffered saline at 4°C and measured fresh within 24 hours of collection. The specimens were placed on top of wet gauze in a 35-mm petri dish and marked with black ink at four corners to facilitate coregistration with histology. The specimens were warmed up in a water bath to 37°. The petri dish containing the specimens was placed in a sample holder connected to a two-axis motorized stage for SPARSE measurement.

### Histological analysis

Following SPARSE imaging, breast tissue specimens were placed in 10% formalin for at least 72 hours and treated with Dissect-aid. The specimens were then embedded in paraffin, sectioned at 100-μm increments in depth (7-μm-thick sections), and stained with H&E and PSR, following routine protocols and Wellman photo pathology core. The H&E slides were digitized using Nano-zoomer whole-slide scanner (2.0 HT, Hamamatsu, Japan). PSR slides were manually digitized using a circularly polarized light microscope (BX43, Nikon, USA, 4x). Fiducial ink marks in the sample photographs and bright-field images were used to coregister the SPARSE maps with histology. A custom MATLAB script was developed to segment H&E-stained slides into major tissue compartments, tumor epithelium, stroma, adipose, and background, using color-space analysis. RGB images were converted to Lab color space, and the color-thresholding App was applied to group pixels based on colorimetric similarity to create region-specific binary masks. These masks were used in a block-wise classification scheme to produce a coarse tissue label map. The histology-derived segments were scaled down to match the SPARSE image dimensions. We then extracted and smoothed region boundaries and overlaid them onto each SPARSE-derived map using consistent color and linestyle conventions. This enabled visual and quantitative comparisons between histological structures and SPARSE.

Wright-Giemsa–stained blood smear slides were digitized using a NanoZoomer whole-slide scanner (2.0 HT, Hamamatsu, Japan). Several randomly selected regions of interest (magnification: 80×, ROI: 1920 × 1080 pixels, pixel size: 0.057 μm) were exported as .jpg image files. A custom MATLAB script was used to sequentially read, threshold, and binarize the images to isolate individual RBCs. This was followed by a connected component analysis to measure projected cell area. The equivalent radii of each cell was then computed as $=\sqrt{A/\pi }$, assuming a circular profile. A histogram of RBC radius distribution, along with the average and SDs, was obtained.

### Dynamic light scattering

The particle size values of polystyrene microsphere suspensions and milk were verified through comparison with DLS measurements (Zeta Sizer Ultra, Malvern Instruments). For polystyrene microspheres, 5 μl of the stock suspension was mixed with 1 ml in a spectroscopic cuvette. In case of milk samples, 100 μl of specimens was mixed with 900 μl of deionized water in the cuvette.

### Statistical analysis

A multiple linear regression model (stepwiselm, MATLAB 2022a), containing forward and backward stepwise regression was used to determine the interdependencies between synthetic *X*_1_, *X*_2_, *X*_3_, and *X*_4_ metrics, identify the most influential individual metrics and interaction terms, and determine the final model for each cluster (cluster #1: *N* = 437, *R*^2^ = 0.723, *P* = 5 × 10^−125^; cluster #2: *N* = 256, *R*^2^ = 0.972, *P* = 5 × 10^−191^; cluster #3: *N* = 531, *R*^2^ = 0.955, *P* = 0; cluster #4: *N* = 1866, *R*^2^ = 0.796, *P* = 0; cluster #5: *N* = 434, *R*^2^ = 0.743, *P* = 1.8 × 10^−122^). Linear regression analysis was performed to compare SPARSE measurements with DLS-based characterizations of polystyrene microsphere and milk particle radii, as well as with smear microscopy image analysis of swine RBC size, as shown in Figs. 3, 4, and 5 (polystyrene microspheres, *N* = 14; milk, *N* = 4; swine blood, *N* = 13). Error bars in the regression plots stand for experimental variations across three different measurements. In addition, the correlation coefficient, *R*, the significance level, *P* value, are reported in the captions of Figs. 3, 4, and 5. In all cases, *P* < 0.05 was considered statistically significant.

## Supplementary Materials

### This PDF file includes:

Supplementary Text Figs. S1 to S10 Tables S1 to S4 References

## References

[1] M. Akkerman, L. B. Johansen, V. Rauh, J. Sørensen, L. B. Larsen, N. A. Poulsen, Relationship between casein micelle size, protein composition and stability of UHT milk. Int. Dairy J. 112, 104856 (2021).

[2] I. S. Pires, J. R. Suggs, I. S. Carlo, D. Yun, P. T. Hammond, D. J. Irvine, Surfactant-mediated assembly of precision-size liposomes. Chem. Mater. 36, 7263–7273 (2024). 39156714 10.1021/acs.chemmater.4c01127 PMC11325547

[3] M. Danaei, M. Dehghankhold, S. Ataei, F. Hasanzadeh Davarani, R. Javanmard, A. Dokhani, S. Khorasani, M. R. Mozafari, Impact of particle size and polydispersity index on the clinical applications of lipidic nanocarrier systems. Pharmaceutics 10, 57 (2018). 29783687 10.3390/pharmaceutics10020057 PMC6027495

[4] P. Jevtić, L. J. Edens, L. D. Vuković, D. L. Levy, Sizing and shaping the nucleus: Mechanisms and significance. Curr. Opin. Cell Biol. 28, 16–27 (2014). 24503411 10.1016/j.ceb.2014.01.003 PMC4061251

[5] J. Stetefeld, S. A. McKenna, T. R. Patel, Dynamic light scattering: A practical guide and applications in biomedical sciences. Biophys. Rev. 8, 409–427 (2016). 28510011 10.1007/s12551-016-0218-6 PMC5425802

[6] G. L. Salvagno, F. Sanchis-Gomar, A. Picanza, G. Lippi, Red blood cell distribution width: A simple parameter with multiple clinical applications. Crit. Rev. Clin. Lab. Sci. 52, 86–105 (2015). 25535770 10.3109/10408363.2014.992064

[7] K. M. Kim, L. Y. Lui, W. S. Browner, J. A. Cauley, K. E. Ensrud, D. M. Kado, E. S. Orwoll, J. T. Schousboe, S. R. Cummings, Association between variation in red cell size and multiple aging-related outcomes. J. Gerontol. A Biol. Sci. Med. Sci. 76, 1288–1294 (2021). 32894755 10.1093/gerona/glaa217 PMC8202142

[8] S. Dixit, T. Jha, R. Gupta, D. Shah, N. Dayal, M. Kotru, Practical approach to the interpretation of complete blood count reports and histograms. Indian Pediatr. 59, 485–491 (2022). 35695142

[9] D. H. Tycko, M. H. Metz, E. A. Epstein, A. Grinbaum, Flow-cytometric light scattering measurement of red blood cell volume and hemoglobin concentration. Appl. Optics 24, 1355 (1985). 10.1364/ao.24.001355 18223719

[10] M. Egeblad, M. G. Rasch, V. M. Weaver, Dynamic interplay between the collagen scaffold and tumor evolution. Curr. Opin. Cell Biol. 22, 697–706 (2010). 20822891 10.1016/j.ceb.2010.08.015 PMC2948601

[11] I. A. Hatton, E. D. Galbraith, N. S. C. Merleau, T. P. Miettinen, B. M. Smith, J. A. Shander, The human cell count and size distribution. Proc. Natl. Acad. Sci. U.S.A. 120, e2303077120 (2023). 37722043 10.1073/pnas.2303077120 PMC10523466

[12] *Light Scattering from Microstructures*. F. Moreno, F. Gozalez, Eds. (Springer, 2000).

[13] Z. Ma, H. G. Merkus, J. G. A. E. de Smet, C. Heffels, B. Scarlett, New developments in particle characterization by laser diffraction: Size and shape. Powder Technol. 111, 66–78 (2000).

[14] C. Lohr, A. H. Kunding, V. K. Bhatia, D. Stamou, in *Methods in Enzymology* (Academic Press, 2009), vol. 465, pp. 143–160. 10.1016/S0076-6879(09)65008-4 19913166

[15] A. H. Kunding, M. W. Mortensen, S. M. Christensen, D. Stamou, A Fluorescence-based technique to construct size distributions from single-object measurements: Application to the extrusion of lipid vesicles. Biophys. J. 95, 1176–1188 (2008). 18424503 10.1529/biophysj.108.128819 PMC2479610

[16] F. Schleinzer, M. Strebl, M. Blech, P. Garidel, Backgrounded membrane imaging—A valuable alternative for particle detection of biotherapeutics? J. Pharm. Innov. 18, 1575–1593 (2023).

[17] S. K. Vargas, A. Eskafi, E. Carter, N. Ciaccio, A comparison of background membrane imaging versus flow technologies for subvisible particle analysis of biologics. Int. J. Pharm. 578, 119072 (2020). 32001293 10.1016/j.ijpharm.2020.119072

[18] K. J. Chalut, J. H. Ostrander, M. G. Giacomelli, A. Wax, Light Scattering measurements of subcellular structure provide noninvasive early detection of chemotherapy-induced apoptosis. Cancer Res. 69, 1199–1204 (2009). 19141640 10.1158/0008-5472.CAN-08-3079 PMC2667891

[19] Z. A. Steelman, D. Ho, K. K. Chu, A. Wax, Scanning system for angle-resolved low-coherence interferometry. Opt. Lett. 42, 4581–4584 (2017). 29140317 10.1364/OL.42.004581 PMC5777518

[20] A. Wax, C. Yang, V. Backman, K. Badizadegan, C. W. Boone, R. R. Dasari, M. S. Feld, Cellular organization and substructure measured using angle-resolved low-coherence interferometry. Biophys. J. 82, 2256–2264 (2002). 11916880 10.1016/S0006-3495(02)75571-9 PMC1302018

[21] Q. Zhang, J. C. Gamekkanda, A. Pandit, W. Tang, C. Papageorgiou, C. Mitchell, Y. Yang, M. Schwaerzler, T. Oyetunde, R. D. Braatz, A. S. Myerson, G. Barbastathis, Extracting particle size distribution from laser speckle with a physics-enhanced autocorrelation-based estimator (PEACE). Nat. Commun. 14, 1159 (2023). 36859392 10.1038/s41467-023-36816-2 PMC9977959

[22] J. W. Goodman, *Speckle Phenomena in Optics: Theory and Applications* (Roberts & Co., 2007), pp. xvi, 387 p.

[23] S. K. Nadkarni, B. E. Bouma, T. Helg, R. Chan, E. Halpern, A. Chau, M. S. Minsky, J. T. Motz, S. L. Houser, G. J. Tearney, Characterization of atherosclerotic plaques by laser speckle imaging. Circulation 112, 885–892 (2005). 16061738 10.1161/CIRCULATIONAHA.104.520098 PMC2957879

[24] Z. Hajjarian, S. K. Nadkarni, Evaluating the viscoelastic properties of tissue from laser speckle fluctuations. Sci. Rep. 2, 316 (2012). 22428085 10.1038/srep00316 PMC3306019

[25] Z. Hajjarian, S. K. Nadkarni, Tutorial on laser speckle rheology: Technology, applications, and opportunities. J. Biomed. Opt. 25, 1–19 (2020). 10.1117/1.JBO.25.5.050801 PMC7195443 32358928

[26] S. K. Nadkarni, A. Bilenca, B. E. Bouma, G. J. Tearney, Measurement of fibrous cap thickness in atherosclerotic plaques by spatiotemporal analysis of laser speckle images. J. Biomed. Opt. 11, 021006 (2006). 16674181 10.1117/1.2186046 PMC2978660

[27] Z. Hajjarian, S. K. Nadkarni, Evaluation and correction for optical scattering variations in laser speckle rheology of biological fluids. PLOS ONE 8, e65014 (2013). 23705028 10.1371/journal.pone.0065014 PMC3660338

[28] Z. Hajjarian, S. K. Nadkarni, Correction of optical absorption and scattering variations in laser speckle rheology measurements. Opt. Express 22, 6349–6361 (2014). 24663983 10.1364/OE.22.006349 PMC4083052

[29] Z. Hajjarian, S. K. Nadkarni, Estimation of particle size variations for laser speckle rheology of materials. Opt. Lett. 40, 764–767 (2015). 25723427 10.1364/OL.40.000764 PMC4605544

[30] F. C. MacKintosh, J. X. Zhu, D. J. Pine, D. A. Weitz, Polarization memory of multiply scattered light. Phys. Rev. B Condens. Matter 40, 9342–9345 (1989). 9991437 10.1103/physrevb.40.9342

[31] D. A. Weitz, D. J. Pine, in *Dynamic Light Scattering: The Method and Some Applications*, W. Brown, Ed. (Oxford Univ. Press, 1993).

[32] Z. Hajjarian, E. F. Brachtel, D. M. Tshikudi, S. K. Nadkarni, Mapping mechanical properties of the tumor microenvironment by laser speckle rheological microscopy. Cancer Res. 81, 4874–4885 (2021). 34526347 10.1158/0008-5472.CAN-20-3898 PMC8524785

[33] W. Hergert, T. Wriedt, *The Mie Theory, Basics and Applications* (Springer2012).

[34] S. L. Jacques, B. W. Pogue, Tutorial on diffuse light transport. J. Biomed. Opt. 13, 041302 (2008). 19021310 10.1117/1.2967535

[35] T. J. Farrell, M. S. Patterson, B. Wilson, A diffusion theory model of spatially resolved, steady-state diffuse reflectance for the noninvasive determination of tissue optical properties in vivo. Med. Phys. 19, 879–888 (1992). 1518476 10.1118/1.596777

[36] A. H. Hielscher, J. R. Mourant, I. J. Bigio, in *Biomedical Optical Spectroscopy and Diagnostics*, E. Sevick-Muraca, D. Benaron, Eds. (Optica Publishing Group, 1996), vol. 3, pp. SP3.

[37] S. L. Jacques, Optical properties of biological tissues: A review. Phys. Med. Biol. 58, R37–R61 (2013). 23666068 10.1088/0031-9155/58/11/R37

[38] A. N. Bashkatov, E. A. Genina, V. I. Kochubey, V. V. Tuchin, Optical properties of human skin, subcutaneous and mucous tissues in the wavelength range from 400 to 2000 nm. J. Phys. D Appl. Phys. 38, 2543–2555 (2005).

[39] M. Johns, C. A. Giller, D. C. German, H. Liu, Determination of reduced scattering coefficient of biological tissue from a needle-like probe. Opt. Express 13, 4828–4842 (2005). 19498468 10.1364/opex.13.004828

[40] F. Poulon, H. Mehidine, M. Juchaux, P. Varlet, B. Devaux, J. Pallud, D. Abi Haidar, Optical properties, spectral, and lifetime measurements of central nervous system tumors in humans. Sci. Rep. 7, 13995 (2017). 29070870 10.1038/s41598-017-14381-1 PMC5656602

[41] A. M. Zysk, S. G. Adie, J. J. Armstrong, M. S. Leigh, A. Paduch, D. D. Sampson, F. T. Nguyen, S. A. Boppart, Needle-based refractive index measurement using low-coherence interferometry. Opt. Lett. 32, 385–387 (2007). 17356661 10.1364/ol.32.000385

[42] L. M. C. Oliveira, V. V. Tuchin, in *The Optical Clearing Method: A New Tool for Clinical Practice and Biomedical Engineering* (Springer International Publishing, 2019), pp. 1–15.

[43] K. Takamura, H. Fischer, N. R. Morrow, Physical properties of aqueous glycerol solutions. J. Petrol. Sci. Eng. 98-99, 50–60 (2012).

[44] M. Glantz, T. G. Devold, G. E. Vegarud, H. Lindmark Månsson, H. Stålhammar, M. Paulsson, Importance of casein micelle size and milk composition for milk gelation. J. Dairy Sci. 93, 1444–1451 (2010). 20338421 10.3168/jds.2009-2856

[45] M. C. Michalski, V. Briard, F. Michel, Optical parameters of milk fat globules for laser light scattering measurements. Lait 81, 787–796 (2001).

[46] M. C. Ambrose Griffin, W. G. Griffin, A simple turbidimetric method for the determination of the refractive index of large colloidal particles applied to casein micelles. J. Colloid Interface Sci. 104, 409–415 (1985).

[47] K. Takahashi, H. Kato, T. Saito, S. Matsuyama, S. Kinugasa, Precise measurement of the size of nanoparticles by dynamic light scattering with uncertainty analysis. Part. Part. Syst. Charact. 25, 31–38 (2008).

[48] N. Bosschaart, G. J. Edelman, M. C. Aalders, T. G. van Leeuwen, D. J. Faber, A literature review and novel theoretical approach on the optical properties of whole blood. Lasers Med. Sci. 29, 453–479 (2014). 24122065 10.1007/s10103-013-1446-7 PMC3953607

[49] L. K. Goodhead, F. M. MacMillan, Measuring osmosis and hemolysis of red blood cells. Adv. Physiol. Educ. 41, 298–305 (2017). 28526694 10.1152/advan.00083.2016

[50] R. Gautam, Y. Xiang, J. Lamstein, Y. Liang, A. Bezryadina, G. Liang, T. Hansson, B. Wetzel, D. Preece, A. White, M. Silverman, S. Kazarian, J. Xu, R. Morandotti, Z. Chen, Optical force-induced nonlinearity and self-guiding of light in human red blood cell suspensions. Light Sci. Appl. 8, 31 (2019). 30886708 10.1038/s41377-019-0142-1 PMC6414597

[51] M. Wojdyla, S. Raj, D. Petrov, Absorption spectroscopy of single red blood cells in the presence of mechanical deformations induced by optical traps. J. Biomed. Opt. 17, 97006–97011 (2012). 23085923 10.1117/1.JBO.17.9.097006

[52] P. Ecker, A. Sparer, B. Lukitsch, M. Elenkov, M. Seltenhammer, R. Crevenna, M. Gföhler, M. Harasek, U. Windberger, Animal blood in translational research: How to adjust animal blood viscosity to the human standard. Physiol. Rep. 9, e14880 (2021). 34042285 10.14814/phy2.14880 PMC8157792

[53] T. Matsuzawa, Y. Ikarashi, Haemolysis of various mammalian erythrocytes in sodium chloride, glucose and phosphate-buffer solutions. Lab. Anim 13, 329–331 (1979). 43414 10.1258/002367779780943297

[54] A. Rezghi, J. Zhang, Tank-treading dynamics of red blood cells in shear flow: On the membrane viscosity rheology. Biophys. J. 121, 3393–3410 (2022). 35986517 10.1016/j.bpj.2022.08.016 PMC9515232

[55] M. Kinnunen, A. Kauppila, A. Karmenyan, R. Myllylä, Effect of the size and shape of a red blood cell on elastic light scattering properties at the single-cell level. Biomed. Opt. Express 2, 1803–1814 (2011). 21750759 10.1364/BOE.2.001803 PMC3130568

[56] J. N. Ouellette, C. R. Drifka, K. B. Pointer, Y. Liu, T. J. Lieberthal, W. J. Kao, J. S. Kuo, A. G. Loeffler, K. W. Eliceiri, Navigating the collagen jungle: The biomedical potential of fiber organization in cancer. Bioengineering 8, 17 (2021). 33494220 10.3390/bioengineering8020017 PMC7909776

[57] M. M. M. Almekinders, M. Schaapveld, B. Thijssen, L. L. Visser, T. Bismeijer, J. Sanders, E. Isnaldi, I. Hofland, M. Mertz, L. F. A. Wessels, A. Broeks, E. Hooijberg, W. Zwart, E. H. Lips, Grand Challenge PRECISION Consortium, C. Desmedt, J. Wesseling, Breast adipocyte size associates with ipsilateral invasive breast cancer risk after ductal carcinoma in situ. NPJ Breast Cancer 7, 31 (2021). 33753731 10.1038/s41523-021-00232-w PMC7985299

[58] M.-K. Hayward, J. Louise Jones, A. Hall, L. King, A. J. Ironside, A. C. Nelson, E. Shelley Hwang, V. M. Weaver, Derivation of a nuclear heterogeneity image index to grade DCIS. Comput. Struct. Biotechnol. J. 18, 4063–4070 (2020). 33363702 10.1016/j.csbj.2020.11.040 PMC7744935

[59] F. Schuh, J. V. Biazús, E. Resetkova, C. Z. Benfica, A. de Freitas Ventura, D. Uchoa, M. Graudenz, M. I. A. Edelweiss, Histopathological grading of breast ductal carcinoma in situ: Validation of a web-based survey through intra-observer reproducibility analysis. Diagn. Pathol. 10, 93 (2015). 26159429 10.1186/s13000-015-0320-2 PMC4702358

[60] R. Hussain, M. Alican Noyan, G. Woyessa, R. R. Retamal Marin, P. Antonio Martinez, F. M. Mahdi, V. Finazzi, T. A. Hazlehurst, T. N. Hunter, T. Coll, M. Stintz, F. Muller, G. Chalkias, V. Pruneri, An ultra-compact particle size analyser using a CMOS image sensor and machine learning. Light Sci. Appl. 9, 21 (2020). 32128161 10.1038/s41377-020-0255-6 PMC7016131

[61] C. S. Mulvey, K. Zhang, W.-H. B. Liu, D. J. Waxman, I. J. Bigio, Wavelength-dependent backscattering measurements for quantitative monitoring of apoptosis, Part 2: Early spectral changes during apoptosis are linked to apoptotic volume decrease. J. Biomed. Opt. 16, 117002 (2011). 22112134 10.1117/1.3644911 PMC3221718

[62] V. Backman, V. Gopal, M. Kalashnikov, K. Badizadegan, R. Gurjar, A. Wax, I. Georgakoudi, M. Mueller, C. W. Boone, R. R. Dasari, M. S. Feld, Measuring cellular structure at submicrometer scale with light scattering spectroscopy. IEEE J. Sel. Top.Quantum Electron. 7, 887–893 (2001).

[63] E. T. Jelly, Z. A. Steelman, H. Zhang, K. K. Chu, C. C. Cotton, S. Eluri, N. J. Shaheen, A. Wax, Next-generation endoscopic probe for detection of esophageal dysplasia using combined OCT and angle-resolved low-coherence interferometry. Biomed. Opt. Express 15, 1943–1958 (2024). 38495690 10.1364/BOE.515469 PMC10942713

[64] C. van Dooijeweert, P. J. van Diest, I. O. Ellis, Grading of invasive breast carcinoma: The way forward. Virchows Arch. 480, 33–43 (2022). 34196797 10.1007/s00428-021-03141-2 PMC8983621

[65] S. C. Wetstein, V. M. T. de Jong, N. Stathonikos, M. Opdam, G. M. H. E. Dackus, J. P. W. Pluim, P. J. van Diest, M. Veta, Deep learning-based breast cancer grading and survival analysis on whole-slide histopathology images. Sci. Rep. 12, 15102 (2022). 36068311 10.1038/s41598-022-19112-9 PMC9448798

[66] R. C. Chan, C. K. C. To, K. C. T. Cheng, T. Yoshikazu, L. L. A. Yan, G. M. Tse, Artificial intelligence in breast cancer histopathology. Histopathology 82, 198–210 (2023). 36482271 10.1111/his.14820

[67] G. Xi, W. Guo, D. Kang, J. Ma, F. Fu, L. Qiu, L. Zheng, J. He, N. Fang, J. Chen, J. Li, S. Zhuo, X. Liao, H. Tu, L. Li, Q. Zhang, C. Wang, S. A. Boppart, J. Chen, Large-scale tumor-associated collagen signatures identify high-risk breast cancer patients. Theranostics 11, 3229–3243 (2021). 33537084 10.7150/thno.55921 PMC7847696

[68] S. Gesta, Y.-H. Tseng, C. R. Kahn, Developmental origin of fat: Tracking obesity to its source. Cell 131, 242–256 (2007). 17956727 10.1016/j.cell.2007.10.004

[69] L. X. Ma, C. C. Wang, J. Y. Tan, Light scattering by densely packed optically soft particle systems, with consideration of the particle agglomeration and dependent scattering. Appl. Opt. 58, 7336–7345 (2019). 31674380 10.1364/AO.58.007336

[70] J. M. Schmitt, G. Kumar, Optical scattering properties of soft tissue: A discrete particle model. Appl. Optics 37, 2788–2797 (1998). 10.1364/ao.37.002788 18273225

[71] S. J. Kirkpatrick, D. D. Duncan, E. M. Wells-Gray, Detrimental effects of speckle-pixel size matching in laser speckle contrast imaging. Opt. Lett. 33, 2886–2888 (2008). 19079481 10.1364/ol.33.002886

[72] L. Kaufman, P. J. Rousseeuw, *Finding Groups in Data: An Introduction to Cluster Analysis* (John Wiley & Sons, 2009).

[73] P. J. Rousseeuw, Silhouettes: A graphical aid to the interpretation and validation of cluster analysis. J. Comput. Appl. Math. 20, 53–65 (1987).

[74] N. Uribe-Patarroyo, A. L. Post, S. Ruiz-Lopera, D. J. Faber, B. E. Bouma, Noise and bias in optical coherence tomography intensity signal decorrelation. OSA Contin. 3, 709–741 (2020). 34085035 10.1364/OSAC.385431 PMC8171193

[75] J. C. Ramella-Roman, S. A. Prahl, S. L. Jacques, Three Monte Carlo programs of polarized light transport into scattering media: Part I. Opt. Express 13, 10392–10405 (2005). 19503254 10.1364/opex.13.010392

[76] J. C. Ramella-Roman, S. A. Prahl, S. L. Jacques, Three Monte Carlo programs of polarized light transport into scattering media: Part II. Opt. Express 13, 10392–10405 (2005). 19503254 10.1364/opex.13.010392

