# Supplementary material for: Laser speckle particle sizer (SPARSE) informs the size distribution of tissue granularities
Source: Sci Adv. 2025 Sep 26;11(39):eadu8577. doi: 10.1126/sciadv.adu8577 (PMC13155485; doi:10.1126/sciadv.adu8577)
Supplement: Supplementary file 1 — Supplementary Text Figs. S1 to S10 Tables S1 to S4 References [file sciadv.adu8577_sm.pdf]

Science Advances
AAAS

Supplementary Materials for

Laser speckle particle sizer (SPARSE) informs the size distribution of tissue granularities

Zeinab Hajjarian *et al.*

Corresponding author: Zeinab Hajjarian, zeinab_hajjarian@uml.edu;
Seemantini K. Nadkarni, snadkarni@mgh.harvard.edu

Sci. Adv. **11**, eadu8577 (2025)
DOI: 10.1126/sciadv.adu8577

This PDF file includes:

Supplementary Text
Figs. S1 to S10
Tables S1 to S4
References

Supplementary Text

Nomenclature and Definitions

a: Particle size.

α(t): Diffusion Exponent, reflects the type of motion characterized by MSD, defined as the log-log slope of MSD: $\alpha(t) = \frac{\partial \log(\langle \Delta r^2(t) \rangle)}{\partial \log(t)}$.

D: Diffusion coefficient of particles in a viscous fluid, defined as $D = \frac{k_B T}{6\pi\eta a}$.

g: Anisotropy Factor [dimensionless], The average cosine of the scattering angle. Values range from −1 (completely backscattered) to +1 (completely forward-scattered); biological tissues typically have g ≈ 0.8–0.95.

$g_{2\parallel}$(t): Co-polarized speckle intensity autocorrelation function, second-order temporal autocorrelation of speckle intensity for parallel polarization.

$g_{2\perp}$(t): Cross-polarized speckle intensity autocorrelation function, second-order temporal autocorrelation of speckle intensity for orthogonal polarization.

G*(ω): Shear viscoelastic modulus of the biological specimen, conventionally defined as the ratio of the applied oscillatory shear stress, $\tau(\omega)$, to the resulting shear strain, $\gamma(\omega)$, $G^*(\omega) = \frac{\tau(\omega)}{\gamma(\omega)}$. In the context of micro-rheology, the shear modulus may be expressed as: $G^*(\omega) = \frac{K_B T}{\pi a j \omega \mathcal{F}(\langle \Delta r^2(t) \rangle)}$, where $\mathcal{F}$ stands for Fourier Transform.

η: Viscosity

$\hat{I}_{||}$: Normalized co-polarized speckle intensity envelope, time-averaged speckle intensity measured under parallel polarization detection.

$\hat{I}_{\perp}$: Normalized cross-polarized speckle intensity envelope, time-averaged speckle intensity measured under orthogonal polarization detection.

k_B: 1.38 $\times 10^{-23}$, Boltzmann Constant.

ℓ*: Transport Mean Free Path [mm], The average distance a photon travels before its direction becomes randomized. Calculated as ℓ* = 1 / ($\mu_a + \mu_s'$), or ℓ* = 1 / μ_s' when absorption is negligible.

λ: Laser wavelength

MSD: Time-dependent mean-square displacement (MSD) of particles, Defined as $\langle \Delta r^2(t) \rangle = \langle |r(t + t_0) - r(t_0)|^2 \rangle$, where *r(t)* denotes the particle's position at time *t*.

μ_a : Absorption Coefficient [mm⁻¹], Probability per unit path length that a photon is absorbed by the medium.

μ_s: Scattering Coefficient [mm⁻¹], Probability per unit path length that a photon undergoes scattering due to refractive index mismatches in tissue structures.

μ_s': Reduced Scattering Coefficient [mm⁻¹], Defined as $\mu_s' = \mu_s(1 - g)$, it accounts for the net angular deflection per scattering event.

n_{rel}: The ratio of the refractive indices of scattering particles and that of their surrounding medium, $n_{rel} = \frac{n_{particles}}{n_{medium}}$, where *n* stands for refractive index.

T: Temperature in degrees Kelvin

X_1: Quantifies the angular variation of $\hat{I}_{||}$, defined as $X_1 = \frac{\hat{I}_{||@0^o}}{\hat{I}_{||@90^o}}$, where, $\hat{I}_{||@0^o}$ and $\hat{I}_{||@90^o}$ represent the radially averaged $\hat{I}_{||}$ at 0° and 90° with respect to horizontal axis, in the annular region between inner and outer circles of the $\hat{I}_{||}$ contour at 30% of its peak intensity.

X_2: Quantifies the circularity of $\hat{I}_{\perp}$, defined as $X_2 = \frac{4\pi A}{P^2}$, where *A* and *P* are the area and the perimeter of the $\hat{I}_{\perp}$ contour at 30% of its peak intensity.

X_3: Quantifies the differential decorrelation rate of $g_{2\perp}$(t) and $g_{2\parallel}$(t), defined as $X_3 = \left.\frac{\log(g_{2\perp}(t))}{\log(g_{2\parallel}(t))}\right|_{t=t_{max}}$ at $t=t_{max}$, where the slopes of the two curves and in turn their vertical separation is maximized.

X_4: Quantifies the radial extent of $\hat{I}_{\perp}$, defined as $X_4=\sqrt{(A/\pi)}$.

Generating synthetic library of polarized speckle attributes and the quantitative metrics

We expanded our previously developed polarized-light correlation transfer Monte-Carlo Ray Tracing (PLCT-MCRT) approach, to simulate first- and second-order statistics of the polarized speckle, namely $\hat{I}_{||}$, $\hat{I}_{\perp}$, $g_{2||}(t)$, and $g_{2\perp}(t)$ in turbid media (*29*). The simulated media exhibited the following size range and optical properties: *a*: 10nm -10 μm, μ_s': 0.5-4 mm^{-1}, and μ_a: 0-70%μ_s'. Refractive indices of the particles and the surrounding medium were 1.59 and 1.45 respectively. This resulted in a relative refractive index mismatch of n_{rel}=1.1. For each *a*, Mie theory calculations were used to adjust the particle concentration to attain the desired μ_s' value. For each combination of [a, $\mu_s'=1\ mm^{-1}$, μ_a/μ_s'], a total of 1 million photons were traced in the medium, as they scattered off from one particle and collided with the next until finally remerged back at the surface. A simple geometric scaling was used to generalize the results to the entire μ_s' range. At each scattering event, Mie theory was used to relate the parallel and perpendicular polarized components of the back-scattered light as a function of the incident light using a matrix with diagonal entries of the S1 and S2 parameters as follows(*33*):

$$\begin{bmatrix} E_{||s} \\ E_{\perp s} \end{bmatrix} = \frac{e^{jk(R-z)}}{-jkR} \begin{bmatrix} S_2(\theta) & 0 \\ 0 & S_1(\theta) \end{bmatrix} \begin{bmatrix} E_{||i} \\ E_{\perp i} \end{bmatrix} \quad \text{(S1)}$$

Here, E_i accounts for the incident field and E_s represents the scattered electric field, where

$$E_i = E_0 e^{-jkz+j\omega} \quad \text{(S2)}$$

In which the E_0 is the magnitude, *k* is the wavenumber, *z* is the traveling direction of the incident beam and $\omega=2\pi/\lambda$ is the angular frequency. S1 and S2 depend on the size parameter *x*, or the ratio of the particle size to wavelength, the complex index of refraction of the particle, and the scattering polar angle, θ, through a series of Riccati-Bessel functions, the spherical Bessel functions, and the spherical Henkel functions(*33*). Accordingly, during each scattering event, the direction of the photon changed in azimuthal, ϕ, and polar angles, θ, based on joint probability distribution function of:

$$P(\theta,\varphi) = I_0 S_{11}(\theta) + S_{12}(\theta)[Q_0 \cos(2\varphi) + U_0 \sin(2\varphi)] \quad \text{(S3)}$$

Here $[I_0, Q_0, U_0, V_0]$ stands for the Stokes vector of the incident photon. The Stokes vector of the photon is updated using the single scattering function, as follows(*27-29, 75, 76*):

$$\begin{bmatrix} I_s \\ Q_s \\ U_s \\ V_s \end{bmatrix} = \frac{1}{k^2R^2} \begin{bmatrix} S_{11} & S_{12} & 0 & 0 \\ S_{12} & S_{22} & 0 & 0 \\ 0 & 0 & S_{33} & S_{34} \\ 0 & 0 & -S_{34} & S_{33} \end{bmatrix} \mathrm{R}(\varphi) \begin{bmatrix} I_i \\ Q_i \\ U_i \\ V_i \end{bmatrix} \quad \text{(S4)}$$

where

$$S_{11} = \frac{1}{2}(|S_2|^2 + |S_1|^2)$$
$$S_{12} = \frac{1}{2}(|S_2|^2 - |S_1|^2)$$
$$S_{33} = \frac{1}{2}(S_2^* S_1 + S_2 S_1^*)$$
$$S_{34} = \frac{1}{2} i(S_1 S_2^* - S_2 S_1^*) \quad \text{(S5)}$$

The distance traveled between the successive scattering events is randomly distributed with an average value of $1/\mu_t$, *where* $\mu_t = \mu_s+\mu_a$, μ_s is the scattering coefficient and μ_a is the absorption coefficient. From these simulations, $\hat{I}_{||}$, $\hat{I}_{\perp}$, $g_{2||}(t)$, and $g_{2\perp}(t)$ are extracted for the said [a, $\mu_s'=1\ mm^{-1}$, μ_a/μ_s'], as (*27-29, 75, 76*):

$$\hat{I}_{||} = \mathrm{IR} + \mathrm{QR} \quad \text{(S6)}$$

$$\hat{I}_{\perp} = \mathrm{IR} - \mathrm{QR} \quad \text{(S7)}$$

$$g_{2||}^{\mathrm{MCRT}}(t) - 1 = \left(\sum_{i=1}^{n} W_i(I_i + Q_i) e^{-\frac{1}{3}k^2\langle \Delta r^2(t)\rangle Y_i}\right)^2 \quad \text{(S8)}$$

$$g_{2\perp}^{MCRT}(t) - 1 = \left(\sum_{i=1}^{n} W_i(I_i - Q_i) e^{-\frac{1}{3}k^2\langle \Delta r^2(t)\rangle Y_i}\right)^2 \quad \text{(S9)}$$

Here, IR and QR are the total values of the first two elements of the stokes vectors on the surface, obtained by spatial binning of the Stokes vectors of the returning photons. In addition, I_i and Q_i are the first two elements of the Stokes vectors of the individual returning photons. Moreover, W_i and Y_i are the energy and momentum transfer of the individual returning photons.

The PLCT-MCRT algorithm was executed for a matrix of particle size values *a*, ranging from 10 nm to 10 µm, μ_s'=1mm^{-1}, and μ_a ranging between 0 and 0.7 mm^{-1}, in 0.1 mm^{-1} increments. A simple geometric scaling yields the photon trajectories for all possible combinations with μ_s'=0.5-4mm^{-1} range.

From this exhaustive set of simulated $\hat{I}_{||}$, $\hat{I}_{\perp}$, $g_{2||}(t)$, and $g_{2\perp}(t)$, we calculated the size-dependent metrics $[X_1, X_2, X_3, X_4]$ for each $[a, \mu_s', \mu_a/\mu_s']$ combination, as depicted in Fig. 1 of the manuscript. We further repeated these simulations by changing the n_{rel} to 1.03 and 1.07, while keeping other variables the same, as depicted in Fig. S1 below.

Accommodating for refractive index variations

To accommodate biological samples of lower refractive index variations such as blood, the PLCT-MCRT simulations was repeated for the same range of a, μ_s', and μ_a and reducing the n_{rel} to 1.07 and 1.03. Supplementary Figures S1 and S2 display the simulated $\hat{I}_{||}$, $\hat{I}_{\perp}$ for a range of particle sizes, a, assuming μ_s'=1, and μ_a=0, and n_{rel} values of 1.1, 1.07, and 1.03, respectively. These additional sets of PLCT-MCRT simulations, and the resulting synthetic libraries of $[X_1, X_2, X_3, X_4]$ metrics were used to develop two additional cluster analysis and particle size estimation equations. Supplementary Figure S3 displays the volume plots of X_1, X_2, X_3, and X_4 variations by the particle size, a, reduced scattering coefficient μ_s', and the normalized absorption coefficient, μ_a / μ_s', as well as their cross sections at $\mu_s' = 1$ mm^{-1}, assuming n_{rel}=1.1, 1.07, and 1.03. In addition, Figure S4 displays the cluster assignment and the scatter diagram of a vs X_1, X_2, X_3, X_4, color-coded with the cluster number. Note that depending on n_{rel}, clusters correspond to different segments of μ_s', μ_a / μ_s', and a space. Also, please note that, one may not fully isolate a, n_{rel}, and μ_s'. This is because n_{rel} and a determine the range of possible values for μ_s'. For instance, if n_{rel} =1.03, μ_s' may not exceed 2 mm^{-1} since for such a low refractive index mismatch, the sample may not exhibit a very strong scattering even at higher scattering particle concentrations. Therefore, the cluster assignments displayed in supplementary Fig. S4 for n_{rel}=1.03 and n_{rel}=1.07 are displayed for μ_s'=0.5-4 mm^{-1} only to remain consistent with the initial particle size estimation equations. The synthetic library of $[X_1, X_2, X_3, X_4]$ metrics was used to develop the cluster analysis and the particle size estimation equation of SPARSE, as detailed later.

Investigating the impact of blood absorption on the metrics.

Supplementary Fig. S5 displays the PLCT-MCRT simulated $\hat{I}_{||}$, $\hat{I}_{\perp}$, for varying μ_a / μ_s', assuming the RBC size a=2.8 μm, its relative refractive index compared to plasma n_{rel}=1.03, and the expected reduced scattering coefficient μ_s' =1 mm^{-1}. These simulations reveal that increased absorption prunes the longer paths, pushes the $\hat{I}_{||}$ to the double-lobed pattern, and shrinks the $\hat{I}_{||}$, $\hat{I}_{\perp}$. Consequently, both X_1 and X_4 are reduced in response to absorption. The simulated $\hat{I}_{||}$, $\hat{I}_{\perp}$ at μ_a / μ_s'=0.3 closely resemble the experimentally evaluated $\hat{I}_{||}$, $\hat{I}_{\perp}$ of isotonic whole blood in Fig. 5 of the manuscript.

Developing the K-means clustering analysis & particle size estimation equations

When attempting to develop particle size estimation equation, we noted that the small and large particles exhibit different dependencies on $[X_1, X_2, X_3, X_4]$. For instance, while in turbid media of smaller size scales and weak scattering, a exhibited an inverse linear relation with X_1, at larger size scales and rich scattering, the relationship was direct and exponential. The variability of the relationships between metrics $[X_1, X_2, X_3, X_4]$ metrics and a across the size scales, and additional dependence on optical properties precluded a single equation to estimate a from these metrics across all the size ranges and for materials of different optical properties. In another words, it appeared that multiple tailored particle size estimation equations govern the relationship between $[X_1, X_2, X_3, X_4]$ metrics and a, depending on the size range and turbidity levels.

For this reason, we employed clustering techniques to identify mutually exclusive regions within the $[X_1, X_2, X_3, X_4]$ metrics and a space, enabling the development of tailored particle size estimation equations for each region. To determine the optimal number and boundaries of these regions, we performed K-means clustering, a widely used method that partitions data into a specified number of clusters by minimizing the variance within each cluster (*72*)K-means is particularly useful for its simplicity and efficiency in handling large datasets, making it well-suited for our objective of identifying distinct regions within the multidimensional space (*72*). To calculate the K-centroids, first the centroids are randomly selected or initialized. Subsequently, each $[X_1, X_2, X_3, X_4]$ point is assigned to the nearest centroid (*72*). The centroids of the clusters are then updated as the mean of all points in the cluster. This process is repeated iteratively until the centroids stabilize or the maximum number of iterations is reached (*72*). The performance of the clustering approach is evaluated using the silhouette score, which measures how well each data point fits within its cluster compared to others. If most points have a high silhouette value, then the clustering solution is appropriate. Our analysis suggested that the silhouette score, is optimized when the space is divided into five clusters. These clusters effectively partitioned the simulated $[X_1, X_2, X_3, X_4]$ space into five distinct regions, each corresponding to a different region in the $[a, \mu_s', \mu_a/\mu_s']$ space.

It is important to note that our cluster analysis is based solely on the synthetic $[X_1, X_2, X_3, X_4]$ vectors, independent of the corresponding $[a, \mu_s', \mu_a/\mu s']$ values used to generate these metrics. Figure S4 presents the color-coded clusters within the $[a, \mu_s', \mu_a/\mu s']$ and $[X_1, X_2, X_3, X_4]$ spaces for various n_{rel} values of 1.03, 1.07, and 1.1.

A few interesting observations can be made by reviewing the regions correspond to the clusters in the $[a, \mu_s', \mu_a/ \mu_s']$ space. For instance, assuming n_{rel}=1.1, as displayed in Fig. S4(C), clusters #1 and #5 both roughly corresponds to turbid media with a<100 nm and μ_a / μ_s' changing in the full 0-0.7 range, with the distinction that in cluster #1 μ_s'<2 mm^{-1} and in cluster#5 μ_s'>2 mm^{-1}. In other words, if cluster analysis of experimentally evaluated $[X_1, X_2, X_3, X_4]$ suggest that the sample belongs to cluster#1, one can readily infer that a<100 nm and that the sample is less turbid.

On the other hand, clusters 2,3, and 4 encompass turbid media with generally larger particles, and varying levels of turbidity. It is to be reiterated that these observations are merely retrospective, and that the k-means clustering was performed on the synthetic library of $[X_1, X_2, X_3, X_4]$ metrics irrespective of simulation variables $[a, \mu_s', \mu_a/\mu_s']$ used to generate the polarized laser speckle attributes. Table S1 lists the cluster centroids for $n_{rel}=1.1$. Subsequently, a step-wise regression analysis was used to obtain the tailored particle size estimation equations specific to each cluster, through adding or removing the metrics and their interaction terms, starting from a constant model. The iterative stepwise approach identified the main effect of each of $[X_1, X_2, X_3, X_4]$ metrics and their interaction terms to yield the most accurate model and minimize the mean square error. When devising the equation, it turned that a linear regression best suited cluster#1, i.e. less turbid media of small particle size. However, the particle size estimation equation was of exponential form for the remaining 4 clusters. The coefficients of particle size estimation equations corresponding to individual clusters, and assuming $n_{rel}=1.1$, are listed in Table S2.

Table S2 entries indicate that the coefficients of the particle size estimation equation, corresponding to the contribution of each of the $[X_1, X_2, X_3, X_4]$ metrics and their interaction are different in each cluster. In other words, for each cluster, the particle size is determined from a different set of metrics and interactions terms. Therefore, while all 4 metrics are used to identifying the cluster #, they are different weighted in the particle size estimations equations of individual clusters, as listed below:

$$Cluster\#1 \quad \hat{a} = 439.69 - 254.97X_1 \tag{S10}$$

$$Cluster\#2 \quad \hat{a} = 10^{3.65+0.97X_1-1.85X_2-0.95X_4+0.6X_1X_4+0.64X_2X_4} \tag{S11}$$

$$Cluster\#3 \quad \hat{a} = 10^{6.52+0.65X_1-6.27X_2-2.18X_4+2.73X_1X_2-0.44X_1X_4+2.53X_2X_4} \tag{S12}$$

$$Cluster\#4 \quad \hat{a} = 10^{\left\{\begin{matrix}-182.73+108.45X_1+151.58X_2+132.09X_3+14.05X_4-83.3X_1X_2- \\ 13.73X_1X_3-15.46X_1X_4-107.67X_2X_3+31.48X_2X_4-31.95X_3X_4\end{matrix}\right\}} \tag{S13}$$

$$Cluster\#5 \quad \hat{a} = 10^{126.28-119.87X_1-123.62X_2-2.29X_3-89.99X_4+120.07X_1X_2+94.05X_2X_4} \tag{S14}$$

Supplementary Sample Preparation: One blue-top tube of whole blood specimen from a healthy donor was obtained from MGH Core hematology lab, using our IRB-approved protocol (MGH IRB#2017P000419). A total of 11 samples were prepared by aliquoting 50 µl of whole blood Eppendorf tubes and spiking them with 10µl of saline with concentrations of 0.9-20%, corresponding to final saline concentrations of 0.9%-4.08% in blood. The samples were then pipetted into a custom 3D-printed chamber, with a clear polycarbonate imaging window. In an isotonic environment, i.e. a medium of the same saline molarity (0.9% concentration), normal human red blood cells (RBCs) were expected to be discoid, with an indented center. Conversely, in a hypertonic solution, osmosis pressure expelled the water from inside of the cell to the extracellular space, causing RBCs to shrink and exhibit a notched, crenated appearance (*49*).

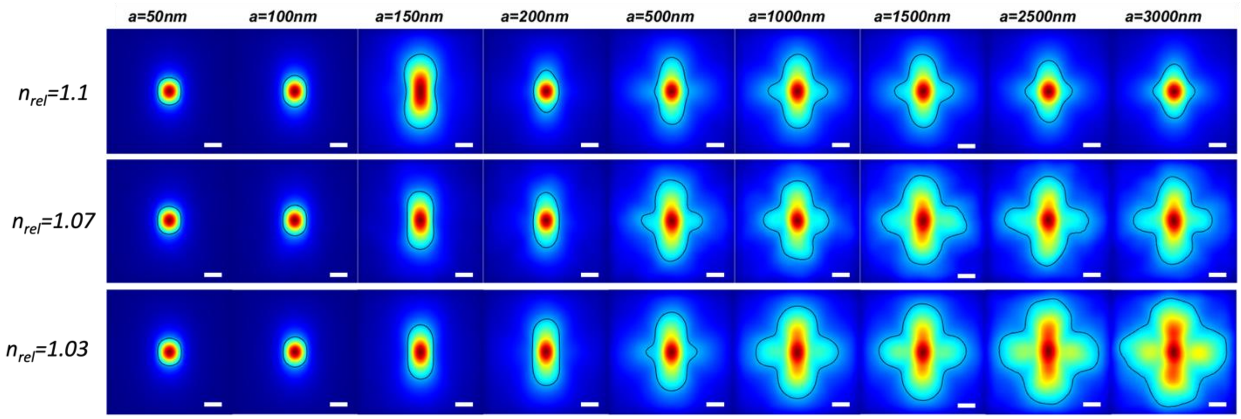

Fig. S1. PLCT-MCRT simulation of $\hat{\boldsymbol{I}}_{||}$, for various relative refractive index mismatch $\boldsymbol{n_{rel}}$. $\hat{\boldsymbol{I}}_{||}$ envelopes are similar for different n_{rel} values as long as a<500 nm. For larger particles, a lower n_{rel} leads to an expanded $\hat{\boldsymbol{I}}_{||}$ with relatively less prominent secondary pair of lobes perpendicular to the polarization axis. Scale bars are 500 μm.

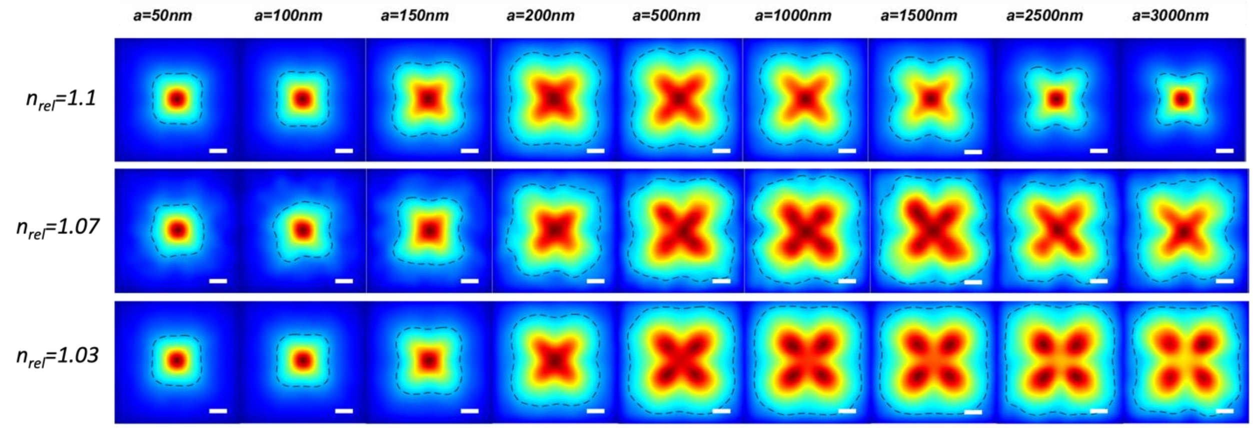

Fig. S2. PLCT-MCRT simulation of $\hat{I}_{\perp}$, for various relative refractive index mismatch n_{rel}. $\hat{I}_{\perp}$ envelopes are similar for different n_{rel} values as long as a<500 nm. For larger particles, a lower n_{rel}, leads to an expanded $\hat{I}_{\perp}$. In addition, while the envelope remails clover-like, its circularity is reduced. Moreover, the intensity maximum shifts from the centroid to the middle of the leaflets. This trend will eventually stop for n_{rel}=1.07 at a>2500nm but continues for n_{rel}=1.03. Scale bars are 500 μm.

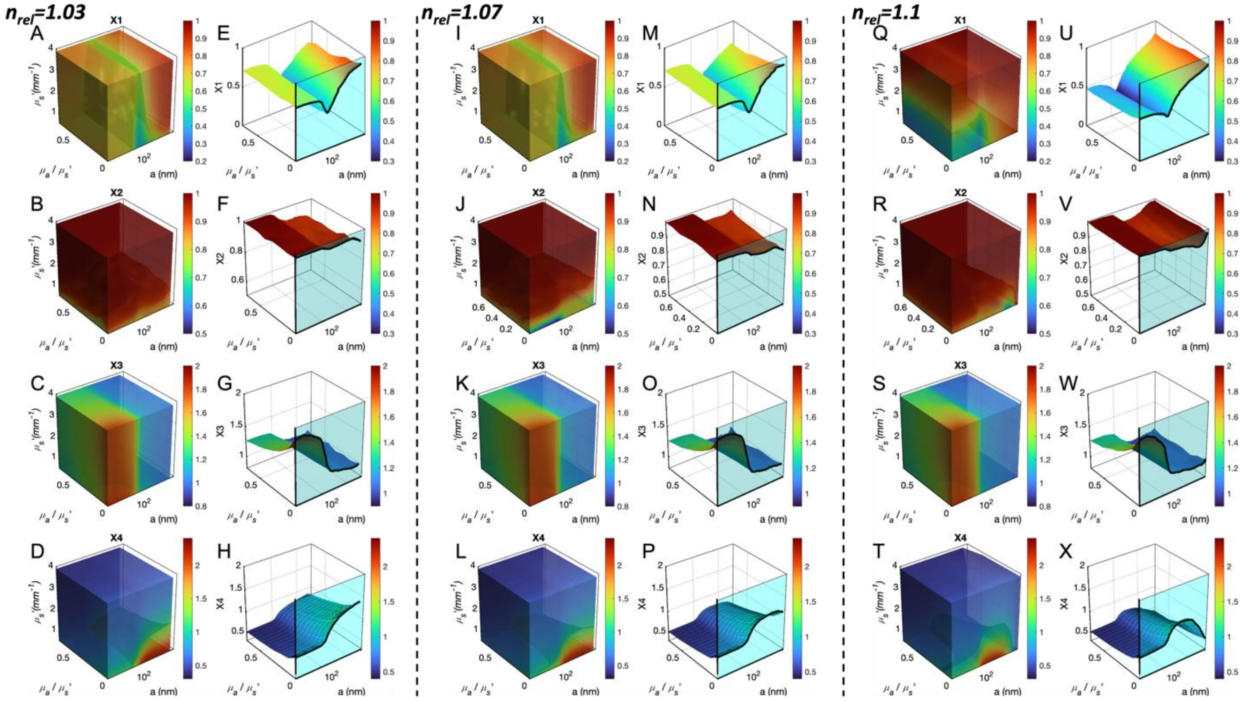

Fig. S3. Synthetic library of size-dependent attributes, created by a Polarized CT-MCRT. (A-D) Volume plots of X_1, X_2, X_3, X_4 variations by the particle size, *a*, reduced scattering coefficient μ_s', and the normalized absorption coefficient, μ_a/μ_s', obtained assuming $n_{rel}=1.03$. (E-H) Cross-section of the volume plots displaying X_1, X_2, X_3, X_4 for a typical $\mu_s'=1\ mm^{-1}$, displaying the variations of the metrics with the particle size, and absorption, obtained assuming $n_{rel}=1.03$. The wall plot, highlighted by a cyan background corresponds to $\mu_s'=1\ mm^{-1}$ and μ_a=0. (I-L) Volume plots of X_1, X_2, X_3, X_4 variations by the particle size, *a*, reduced scattering coefficient μ_s', and the normalized absorption coefficient, μ_a/μ_s', obtained assuming n_{rel}=1.07. (M-P) Cross-section of the volume plots displaying X_1, X_2, X_3, X_4 for a typical $\mu_s'=1\ mm^{-1}$, displaying the variations of the metrics with the particle size, and absorption, obtained assuming $n_{rel}=1.07$. The wall plot, highlighted by a cyan background corresponds to μ_s'=1 mm^{-1} and μ_a=0. (Q-T) Volume plots of X_1, X_2, X_3, X_4 variations by the particle size, a, reduced scattering coefficient μ_s', and the normalized absorption coefficient, μ_a/μ_s', obtained assuming n_{rel}=1.1. (U-X) Cross-section of the volume plots displaying X_1, X_2, X_3, X_4 for a typical μ_s'=1 mm^{-1}, displaying the variations of the metrics with the particle size, and absorption, obtained assuming n_{rel}=1.1. The wall plot, highlighted by a cyan background corresponds to μ_s'=1 mm^{-1} and μ_a=0.

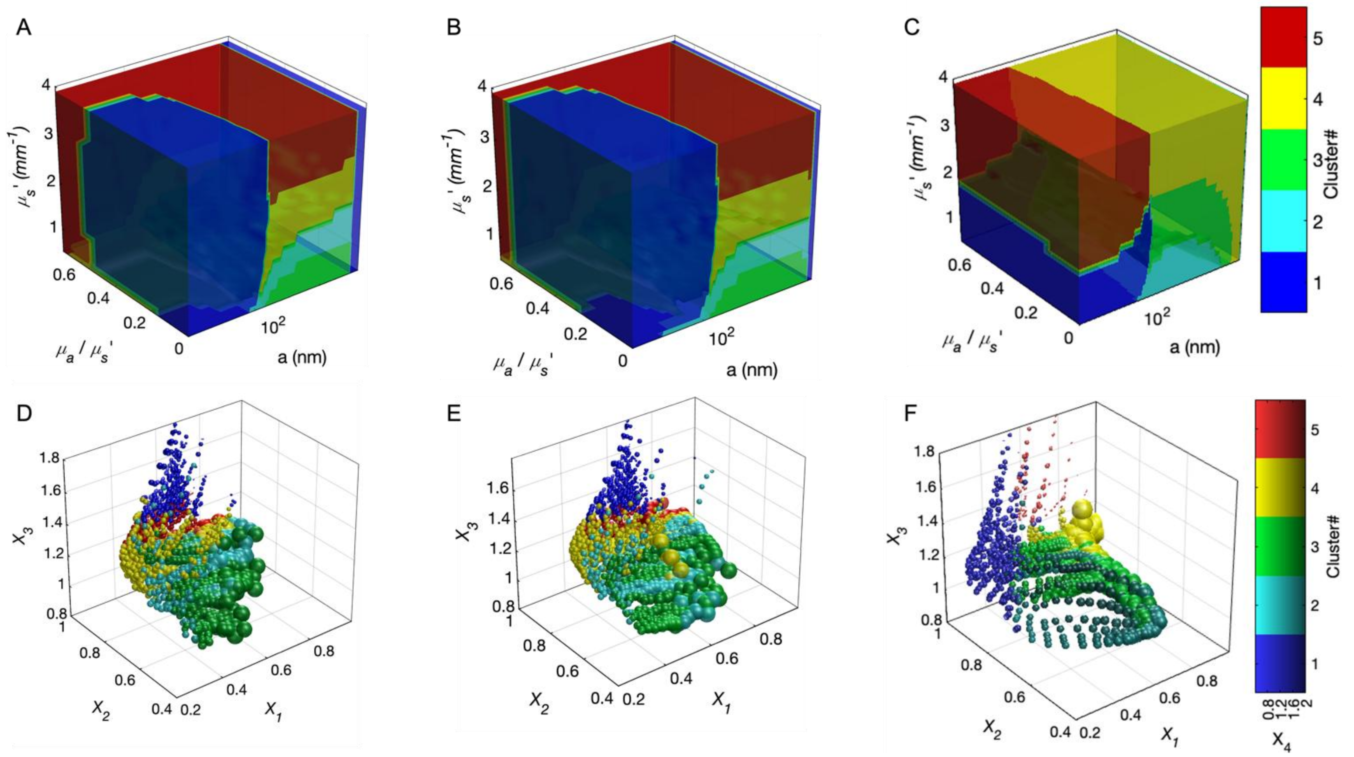

Fig. S4. Cluster analysis of speckle metrics and scatter plots of particle size vs metrics for various nrel. (A-C) Cluster analysis of *[X_1, X_2, X_3, X_4]* metrics, obtained assuming n_{rel} =1.03, 1.07, and 1.1, respectively. The clusters partition the *[a, μ_s', μ_a/μ_s']* space to 5 distinct clusters. (D-F) Scatter plot of the cluster assignments in the *[X_1, X_2, X_3, X_4]* space, obtained assuming n_{rel} =1.03, 1.07, and 1.1, respectively. The 3 axes of the plot correspond to X_1, X_2, and X_3, while X_4 is depicted by varying the luminescence of the cluster color. The sizes of spherical markers are proportionate to the particle volume, i.e. $\sqrt[3]{\boldsymbol{a}}$. A tailored particle size estimation equation that formulates a as a function of X_1, X_2, X_3, and X_4 is obtained for each cluster, and each n_{rel}.

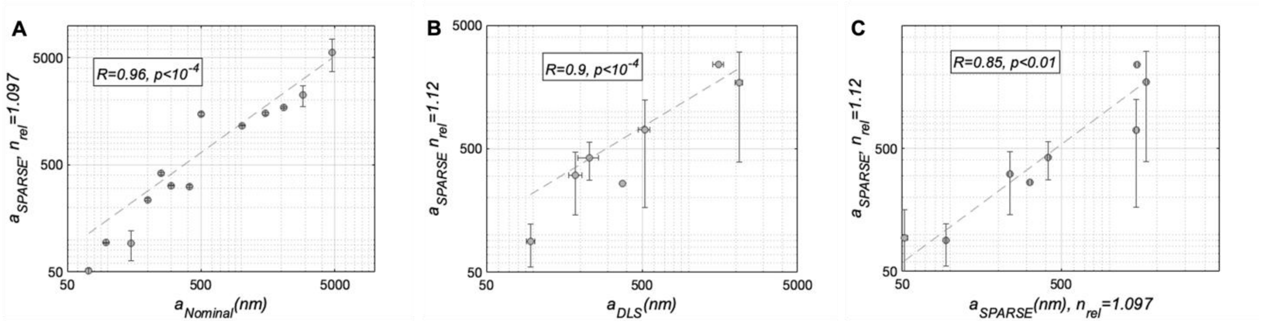

Fig. S5. Validation of SPARSE particle sizing across n_{rel} ≈ 1.09–1.12 using polystyrene microspheres.
(A) Scatter diagram of particle radius, *a*, predicted by SPARSE shows strong, statistically significant correlation with nominal manufacturer-provided values (R = 0.96, $p < 10^{-4}$). **(B)** Scatter diagram of SPARSE-predicted particle radius in specimens of n_{rel}=1.12 vs DLS measurements exhibits strong correlation (R= 0.9, $p < 10^{-4}$). **(C)** SPARSE predictions across microsphere suspensions in media of different refractive indices (n_{rel} = 1.12 vs 1.097) also show strong agreement (R = 0.85, $p < 10^{-2}$.

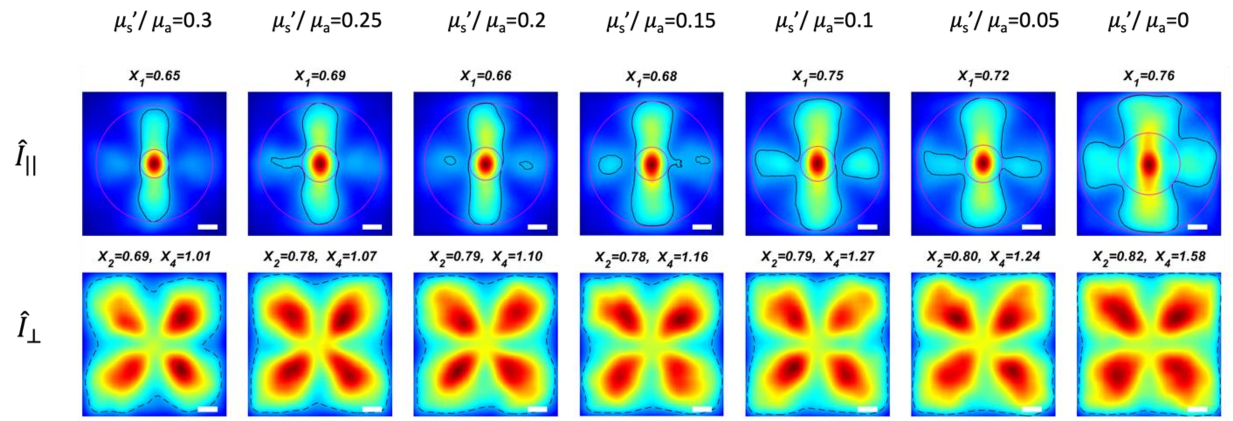

Fig. S6. PLCT-MCRT simulation of $\hat{\boldsymbol{I}}_{||}$ and $\hat{\boldsymbol{I}}_{\perp}$ assuming n_{rel}=1.3, and varying μ_a/μ_s' ratios. Increased absorption reduces the prominence of secondary pair of lobes perpendicular to the polarization axis in $\hat{\boldsymbol{I}}_{||}$, casuing it to transform from a four-leaved pattern to the double-lobed form. In addition, increased absorption causes both $\hat{\boldsymbol{I}}_{||}$ and $\hat{\boldsymbol{I}}_{\perp}$ to shrink, radially. Scale bars are 500 μm.

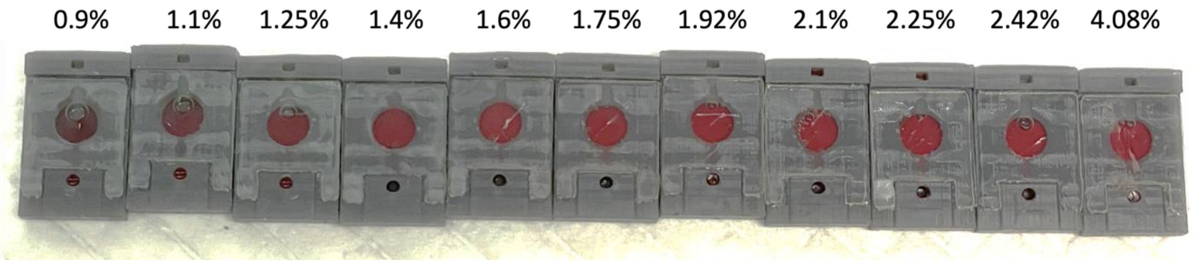

Fig. S7. Human whole blood specimen, spiked with saline solutions of different solute concentrations. As the salt concentration increases, the red color becomes increasingly brighter and more vibrant, indicating reduced absorption and increased scattering.

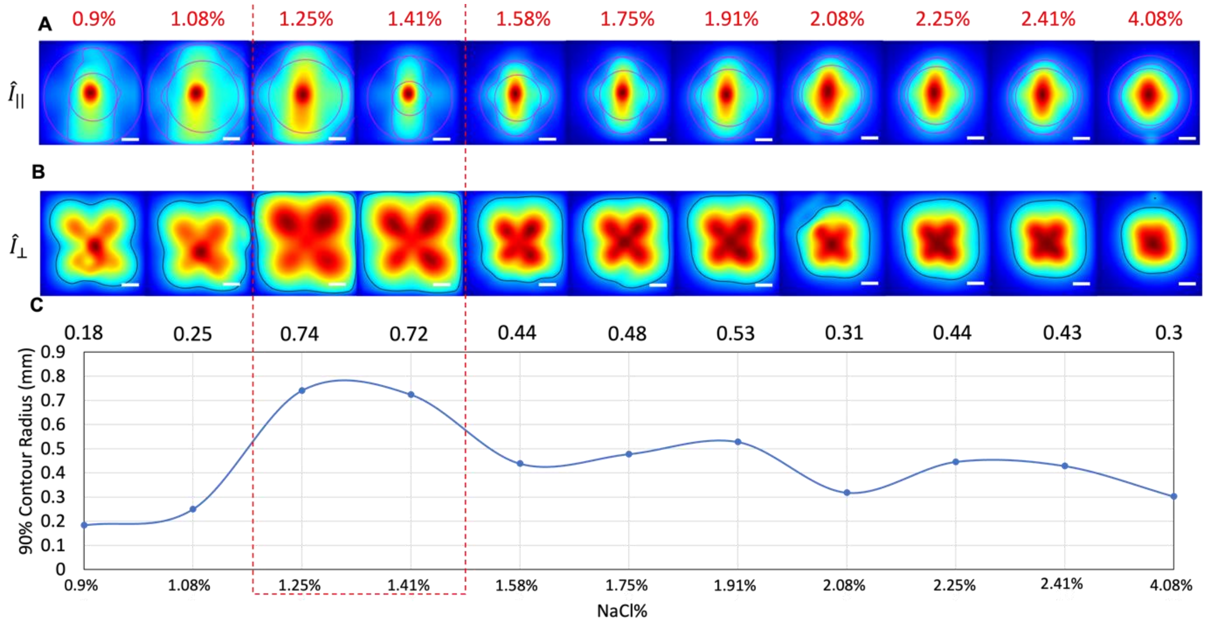

Fig. S8. Experimentally evaluated $\hat{I}_{||}$ (first row) and $\hat{I}_{\perp}$ (second row) of human blood specimens, spiked with saline buffers of varying NaCl concentrations. Also included is the radial location of the $\hat{I}_{\perp}$ peak intensity. It is noted that the maximum intensity is shifted from the centroid to the middle of the leaflets. This shift is quantified through calculating the average radial distance of the region corresponding to the 90th percentile intensity from the centroid of $\hat{I}_{\perp}$. This shift is most prominent for the final NaCl concentrations of 1.25% and 1.41% (red dashed box). Scale bars are 500 μm.

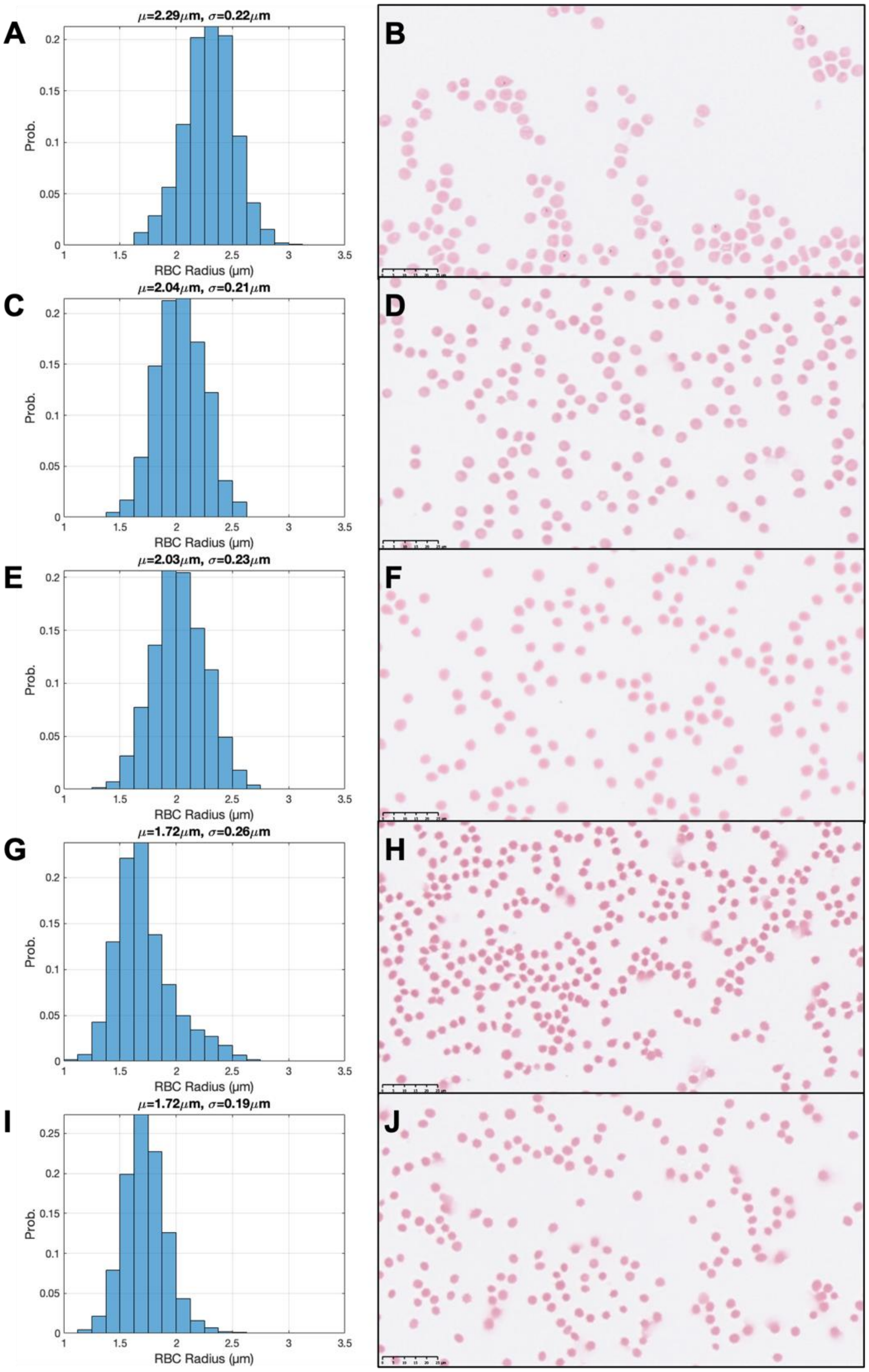

Fig. S9. RBC size distribution along with the corresponding Giemsa-stained smears of swine blood spiked with saline solutions of different NaCl concentrations. (A, B) 0.9%, (C, D)1.1%, (E, F)1.4%, (G, H)1.75%, and (I, J) 2.1%. Visual inspection indicates a progressive reduction in RBCs size with increasing NaCl concentrations. At higher NaCl concentrations (≥1.75%) ghost cells began to appear due to membrane rupture and lysis at higher saline concentrations. Magnification is 80× and scale bars are 25μm.

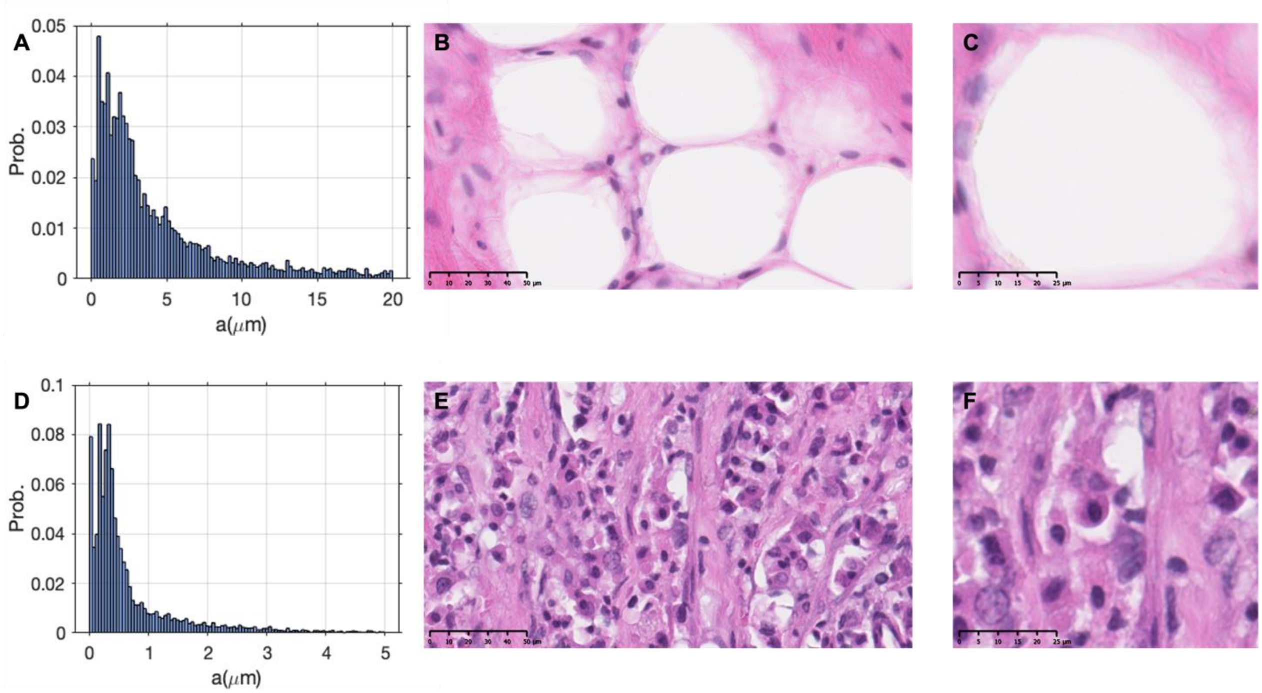

Fig. S10. Probability distribution function of particle size along with high magnification representative H&E slides in soft tissue. (A, D) Histogram of particle size, along with (B, C) 40× (scale bar 50μm) , and (C, F) 80× (scale bar 25 μm)H&E stained histological representation of benign fibroadipose and invasive carcinoma human breast tissue specimens. Normal fibroadipose tissue exhibits a broader distributions stretched towards 10s μms, consistent with the presence of adipocytes cells, which typically range in 10-150 μm radius in humans (12). Conversely, breast carcinoma histogram features a few peaks in the submicron range, and the sizes remain below 5μm, likely reflecting the dense packing of tumor epithelial cells, which typically exhibit high nuclear-to-cytoplasmic ratios and organelle-rich cytoplasm.

**Cluster#**	**X_1**	**X_2**	**X_3**	**X_4**
1	0.43	0.94	1.36	0.79
2	0.61	0.71	1.05	1.85
3	0.68	0.84	1.05	1.08
4	0.89	0.99	1.06	0.58
5	0.84	1.0	1.53	0.52

Table S1. K-centroids obtained by k-mean clustering of synthetic [X_1, X_2, X_3, X_4] metrics. Clusters #1 and #5 represent turbid media with a<100 nm, differentiated by μ_s' values (<2 mm^{-1} for cluster #1, >2 mm^{-1} for cluster #5). Clusters #2, #3, and #4 exhibit increasingly larger particles and higher turbidity, reflecting progressive changes in particle size, as well as scattering and absorption properties."

Cluster #	Intercept	X_1	X_2	X_3	X_4	$X_1 \times X_2$	$X_1 \times X_3$	$X_1 \times X_4$	$X_2 \times X_3$	$X_2 \times X_4$	$X_3 \times X_4$
1	439.69	0	0	-254.97	0	0	0	0	0	0	0
2	3.65	0.97	-1.85	0	-0.95	0	0	0.60	0	0.64	0
3	6.52	0.65	-6.27	0	-2.18	2.73	0	-0.44	0	2.53	0
4	-182.73	108.45	151.58	132.09	14.05	-83.30	-13.73	-15.46	-107.67	31.48	-31.95
5	126.28	-119.87	-123.62	-2.29	-89.99	120.07	0	0	0	94.05	0

Table S2. The coefficient of particle size estimation equation for each cluster, showing the contributions of individual metrics *[X_1,X_2,X_3,X_4]* and their interaction terms. Each cluster uses a distinct set of metrics and interaction weights for particle size determination. While all four metrics contribute to cluster identification, their weights vary across particle size estimation equations for individual clusters.

a (nm) (nominal)	μ_{DLS} *(nm)*	σ_{DLS} *(nm)*	μ_{SPARSE} *(nm)*	σ_{SPARSE} *(nm)*	μ_s' (mm^{-1})
72.5	*72.96*	*3.94*	*51.47*	*2.74*	*0.77*
97.5	*95.95*	*5.87*	*93.95*	*1.21*	*1.08*
149	*143.33*	*9.21*	*92.27*	*28.24*	*0.96*
199	*187.58*	*18.7*	*236.69*	*5.43*	*1.07*
251	*229.02*	*35.0*	*414.42*	*9.25*	*0.97*
299	*236.56*	*41.54*	*321.56*	*4.42*	*0.92*
408	*359.97*	*33.85*	*315.71*	*8.17*	*0.81*
502	*519.58*	*45.04*	*1482.99*	*44.13*	*3.05*
1010	*1211.7*	*163.51*	*1159.53*	*8.11*	*2.06*
1505	*1545.4*	*115.60*	*1500.36*	*34.20*	*1.62*
2090	*2098.7*	*111.93*	*1712.23*	*32.39*	*1.3*
2900	*2778.7*	*116.85*	*2235.57*	*481.58*	*1.07*
4800	*4677.7*	*763.49*	*5575.44*	*1852.27*	*0.63*

Table S3 Nominal value of polystyrene microsphere radii value, as characterized by the manufacturer, together with our SPARSE and DLS size measurements. For SPARSE and DLS, values indicate the average size: μ and standard deviation: σ, over N=3 measurements. The table also includes the theoretically calculated μ_s' values.

NaCl%	SPARSE(μm)	SMEAR (μm)
0.9	2.20±0.19	2.29±0.23
1.08	2.51±0.22	2.04±0.21
1.41	1.63±0.43	2.03±0.23
1.75	1.01±0.31	1.72±0.26
2.1	1.00±0.14	1.72±0.19

Table S4 Comparison of RBC size measurements in swine blood of different plasma tonicity using SPARSE and smear microscopy. Each entry represents the average ± standard deviation of red blood cell (RBC) radius under increasing saline concentrations, corresponding to hyperosmotic stress. Both SPARSE and smear microscopy indicate a reduction in RBC radii and size variability with saline concentration.

REFERENCES AND NOTES

1. M. Akkerman, L. B. Johansen, V. Rauh, J. Sørensen, L. B. Larsen, N. A. Poulsen, Relationship between casein micelle size, protein composition and stability of UHT milk. *Int. Dairy J.* **112**, 104856 (2021).

2. I. S. Pires, J. R. Suggs, I. S. Carlo, D. Yun, P. T. Hammond, D. J. Irvine, Surfactant-mediated assembly of precision-size liposomes. *Chem. Mater.* **36**, 7263–7273 (2024).

3. M. Danaei, M. Dehghankhold, S. Ataei, F. Hasanzadeh Davarani, R. Javanmard, A. Dokhani, S. Khorasani, M. R. Mozafari, Impact of particle size and polydispersity index on the clinical applications of lipidic nanocarrier systems. *Pharmaceutics* **10**, 57 (2018).

4. P. Jevtić, L. J. Edens, L. D. Vuković, D. L. Levy, Sizing and shaping the nucleus: Mechanisms and significance. *Curr. Opin. Cell Biol.* **28**, 16–27 (2014).

5. J. Stetefeld, S. A. McKenna, T. R. Patel, Dynamic light scattering: A practical guide and applications in biomedical sciences. *Biophys. Rev.* **8**, 409–427 (2016).

6. G. L. Salvagno, F. Sanchis-Gomar, A. Picanza, G. Lippi, Red blood cell distribution width: A simple parameter with multiple clinical applications. *Crit. Rev. Clin. Lab. Sci.* **52**, 86–105 (2015).

7. K. M. Kim, L. Y. Lui, W. S. Browner, J. A. Cauley, K. E. Ensrud, D. M. Kado, E. S. Orwoll, J. T. Schousboe, S. R. Cummings, Association between variation in red cell size and multiple aging-related outcomes. *J. Gerontol. A Biol. Sci. Med. Sci.* **76**, 1288–1294 (2021).

8. S. Dixit, T. Jha, R. Gupta, D. Shah, N. Dayal, M. Kotru, Practical approach to the interpretation of complete blood count reports and histograms. *Indian Pediatr.* **59**, 485–491 (2022).

9. D. H. Tycko, M. H. Metz, E. A. Epstein, A. Grinbaum, Flow-cytometric light scattering measurement of red blood cell volume and hemoglobin concentration. *Appl. Optics* **24**, 1355 (1985).

10. M. Egeblad, M. G. Rasch, V. M. Weaver, Dynamic interplay between the collagen scaffold and tumor evolution. *Curr. Opin. Cell Biol.* **22**, 697–706 (2010).

11. I. A. Hatton, E. D. Galbraith, N. S. C. Merleau, T. P. Miettinen, B. M. Smith, J. A. Shander, The human cell count and size distribution. *Proc. Natl. Acad. Sci. U.S.A.* **120**, e2303077120 (2023).

12. *Light Scattering from Microstructures*. F. Moreno, F. Gozalez, Eds. (Springer, 2000).

13. Z. Ma, H. G. Merkus, J. G. A. E. de Smet, C. Heffels, B. Scarlett, New developments in particle characterization by laser diffraction: Size and shape. *Powder Technol.* **111**, 66–78 (2000).

14. C. Lohr, A. H. Kunding, V. K. Bhatia, D. Stamou, in *Methods in Enzymology* (Academic Press, 2009), vol. **465**, pp. 143–160.

15. A. H. Kunding, M. W. Mortensen, S. M. Christensen, D. Stamou, A Fluorescence-based technique to construct size distributions from single-object measurements: Application to the extrusion of lipid vesicles. *Biophys. J.* **95**, 1176–1188 (2008).

16. F. Schleinzer, M. Strebl, M. Blech, P. Garidel, Backgrounded membrane imaging—A valuable alternative for particle detection of biotherapeutics? *J. Pharm. Innov.* **18**, 1575–1593 (2023).

17. S. K. Vargas, A. Eskafi, E. Carter, N. Ciaccio, A comparison of background membrane imaging versus flow technologies for subvisible particle analysis of biologics. *Int. J. Pharm.* **578**, 119072 (2020).

18. K. J. Chalut, J. H. Ostrander, M. G. Giacomelli, A. Wax, Light Scattering measurements of subcellular structure provide noninvasive early detection of chemotherapy-induced apoptosis. *Cancer Res.* **69**, 1199–1204 (2009).

19. Z. A. Steelman, D. Ho, K. K. Chu, A. Wax, Scanning system for angle-resolved low-coherence interferometry. *Opt. Lett.* **42**, 4581–4584 (2017).

20. A. Wax, C. Yang, V. Backman, K. Badizadegan, C. W. Boone, R. R. Dasari, M. S. Feld, Cellular organization and substructure measured using angle-resolved low-coherence interferometry. *Biophys. J.* **82**, 2256–2264 (2002).

21. Q. Zhang, J. C. Gamekkanda, A. Pandit, W. Tang, C. Papageorgiou, C. Mitchell, Y. Yang, M. Schwaerzler, T. Oyetunde, R. D. Braatz, A. S. Myerson, G. Barbastathis, Extracting particle size distribution from laser speckle with a physics-enhanced autocorrelation-based estimator (PEACE). *Nat. Commun.* **14**, 1159 (2023).

22. J. W. Goodman, *Speckle Phenomena in Optics : Theory and Applications* (Roberts & Co., 2007), pp. xvi, 387 p.

23. S. K. Nadkarni, B. E. Bouma, T. Helg, R. Chan, E. Halpern, A. Chau, M. S. Minsky, J. T. Motz, S. L. Houser, G. J. Tearney, Characterization of atherosclerotic plaques by laser speckle imaging. *Circulation* **112**, 885–892 (2005).

24. Z. Hajjarian, S. K. Nadkarni, Evaluating the viscoelastic properties of tissue from laser speckle fluctuations. *Sci. Rep.* **2**, 316 (2012).

25. Z. Hajjarian, S. K. Nadkarni, Tutorial on laser speckle rheology: Technology, applications, and opportunities. *J. Biomed. Opt.* **25**, 1–19 (2020).

26. S. K. Nadkarni, A. Bilenca, B. E. Bouma, G. J. Tearney, Measurement of fibrous cap thickness in atherosclerotic plaques by spatiotemporal analysis of laser speckle images. *J. Biomed. Opt.* **11**, 021006 (2006).

27. Z. Hajjarian, S. K. Nadkarni, Evaluation and correction for optical scattering variations in laser speckle rheology of biological fluids. *PLOS ONE* **8**, e65014 (2013).

28. Z. Hajjarian, S. K. Nadkarni, Correction of optical absorption and scattering variations in laser speckle rheology measurements. *Opt. Express* **22**, 6349–6361 (2014).

29. Z. Hajjarian, S. K. Nadkarni, Estimation of particle size variations for laser speckle rheology of materials. *Opt. Lett.* **40**, 764–767 (2015).

30. F. C. MacKintosh, J. X. Zhu, D. J. Pine, D. A. Weitz, Polarization memory of multiply scattered light. *Phys. Rev. B Condens. Matter* **40**, 9342–9345 (1989).

31. D. A. Weitz, D. J. Pine, in *Dynamic Light Scattering: The Method and Some Applications*, W. Brown, Ed. (Oxford Univ. Press, 1993).

32. Z. Hajjarian, E. F. Brachtel, D. M. Tshikudi, S. K. Nadkarni, Mapping mechanical properties of the tumor microenvironment by laser speckle rheological microscopy. *Cancer Res.* **81**, 4874–4885 (2021).

33. W. Hergert, T. Wriedt, *The Mie Theory, Basics and Applications* (Springer2012).

34. S. L. Jacques, B. W. Pogue, Tutorial on diffuse light transport. *J. Biomed. Opt.* **13**, 041302 (2008).

35. T. J. Farrell, M. S. Patterson, B. Wilson, A diffusion theory model of spatially resolved, steady-state diffuse reflectance for the noninvasive determination of tissue optical properties in vivo. *Med. Phys.* **19**, 879–888 (1992).

36. A. H. Hielscher, J. R. Mourant, I. J. Bigio, in *Biomedical Optical Spectroscopy and Diagnostics*, E. Sevick-Muraca, D. Benaron, Eds. (Optica Publishing Group, 1996), vol. 3, pp. SP3.

37. S. L. Jacques, Optical properties of biological tissues: A review. *Phys. Med. Biol.* **58**, R37–61 (2013).

38. A. N. Bashkatov, E. A. Genina, V. I. Kochubey, V. V. Tuchin, Optical properties of human skin, subcutaneous and mucous tissues in the wavelength range from 400 to 2000 nm. *J. Phys. D Appl. Phys.* **38**, 2543–2555 (2005).

39. M. Johns, C. A. Giller, D. C. German, H. Liu, Determination of reduced scattering coefficient of biological tissue from a needle-like probe. *Opt. Express* **13**, 4828–4842 (2005).

40. F. Poulon, H. Mehidine, M. Juchaux, P. Varlet, B. Devaux, J. Pallud, D. Abi Haidar, Optical properties, spectral, and lifetime measurements of central nervous system tumors in humans. *Sci. Rep.* **7**, 13995 (2017).

41. A. M. Zysk, S. G. Adie, J. J. Armstrong, M. S. Leigh, A. Paduch, D. D. Sampson, F. T. Nguyen, S. A. Boppart, Needle-based refractive index measurement using low-coherence interferometry. *Opt. Lett.* **32**, 385–387 (2007).

42. L. M. C. Oliveira, V. V. Tuchin, in *The Optical Clearing Method: A New Tool for Clinical Practice and Biomedical Engineering* (Springer International Publishing, 2019), pp. 1–15.

43. K. Takamura, H. Fischer, N. R. Morrow, Physical properties of aqueous glycerol solutions. *J. Petrol. Sci. Eng.* **98-99**, 50–60 (2012).

44. M. Glantz, T. G. Devold, G. E. Vegarud, H. Lindmark Månsson, H. Stålhammar, M. Paulsson, Importance of casein micelle size and milk composition for milk gelation. *J. Dairy Sci.* **93**, 1444–1451 (2010).

45. M. C. Michalski, V. Briard, F. Michel, Optical parameters of milk fat globules for laser light scattering measurements. *Lait* **81**, 787–796 (2001).

46. M. C. Ambrose Griffin, W. G. Griffin, A simple turbidimetric method for the determination of the refractive index of large colloidal particles applied to casein micelles. *J. Colloid Interface Sci.* **104**, 409–415 (1985).

47. K. Takahashi, H. Kato, T. Saito, S. Matsuyama, S. Kinugasa, Precise measurement of the size of nanoparticles by dynamic light scattering with uncertainty analysis. *Part. Part. Syst. Charact.* **25**, 31–38 (2008).

48. N. Bosschaart, G. J. Edelman, M. C. Aalders, T. G. van Leeuwen, D. J. Faber, A literature review and novel theoretical approach on the optical properties of whole blood. *Lasers Med. Sci.* **29**, 453–479 (2014).

49. L. K. Goodhead, F. M. MacMillan, Measuring osmosis and hemolysis of red blood cells. *Adv. Physiol. Educ.* **41**, 298–305 (2017).

50. R. Gautam, Y. Xiang, J. Lamstein, Y. Liang, A. Bezryadina, G. Liang, T. Hansson, B. Wetzel, D. Preece, A. White, M. Silverman, S. Kazarian, J. Xu, R. Morandotti, Z. Chen, Optical force-induced nonlinearity and self-guiding of light in human red blood cell suspensions. *Light Sci. Appl.* **8**, 31 (2019).

51. M. Wojdyla, S. Raj, D. Petrov, Absorption spectroscopy of single red blood cells in the presence of mechanical deformations induced by optical traps. *J. Biomed. Opt.* **17**, 97006–97001 (2012).

52. P. Ecker, A. Sparer, B. Lukitsch, M. Elenkov, M. Seltenhammer, R. Crevenna, M. Gföhler, M. Harasek, U. Windberger, Animal blood in translational research: How to adjust animal blood viscosity to the human standard. *Physiol. Rep.* **9**, e14880 (2021).

53. T. Matsuzawa, Y. Ikarashi, Haemolysis of various mammalian erythrocytes in sodium chloride, glucose and phosphate-buffer solutions. *Lab. Anim* **13**, 329–331 (1979).

54. A. Rezghi, J. Zhang, Tank-treading dynamics of red blood cells in shear flow: On the membrane viscosity rheology. *Biophys. J.* **121**, 3393–3410 (2022).

55. M. Kinnunen, A. Kauppila, A. Karmenyan, R. Myllylä, Effect of the size and shape of a red blood cell on elastic light scattering properties at the single-cell level. *Biomed. Opt. Express* **2**, 1803–1814 (2011).

56. J. N. Ouellette, C. R. Drifka, K. B. Pointer, Y. Liu, T. J. Lieberthal, W. J. Kao, J. S. Kuo, A. G. Loeffler, K. W. Eliceiri, Navigating the collagen jungle: The biomedical potential of fiber organization in cancer. *Bioengineering* **8**, 17 (2021).

57. M. M. M. Almekinders, M. Schaapveld, B. Thijssen, L. L. Visser, T. Bismeijer, J. Sanders, E. Isnaldi, I. Hofland, M. Mertz, L. F. A. Wessels, A. Broeks, E. Hooijberg, W. Zwart, E. H. Lips, Grand Challenge PRECISION Consortium, C. Desmedt, J. Wesseling, Breast adipocyte size

associates with ipsilateral invasive breast cancer risk after ductal carcinoma in situ. *NPJ Breast Cancer* **7**, 31 (2021).

58. M.-K. Hayward, J. Louise Jones, A. Hall, L. King, A. J. Ironside, A. C. Nelson, E. Shelley Hwang, V. M. Weaver, Derivation of a nuclear heterogeneity image index to grade DCIS. *Comput. Struct. Biotechnol. J.* **18**, 4063–4070 (2020).

59. F. Schuh, J. V. Biazús, E. Resetkova, C. Z. Benfica, A. de Freitas Ventura, D. Uchoa, M. Graudenz, M. I. A. Edelweiss, Histopathological grading of breast ductal carcinoma in situ: Validation of a web-based survey through intra-observer reproducibility analysis. *Diagn. Pathol.* **10**, 93 (2015).

60. R. Hussain, M. Alican Noyan, G. Woyessa, R. R. Retamal Marin, P. Antonio Martinez, F. M. Mahdi, V. Finazzi, T. A. Hazlehurst, T. N. Hunter, T. Coll, M. Stintz, F. Muller, G. Chalkias, V. Pruneri, An ultra-compact particle size analyser using a CMOS image sensor and machine learning. *Light Sci. Appl.* **9**, 21 (2020).

61. C. S. Mulvey, K. Zhang, W.-H. B. Liu, D. J. Waxman, I. J. Bigio, Wavelength-dependent backscattering measurements for quantitative monitoring of apoptosis, Part 2: Early spectral changes during apoptosis are linked to apoptotic volume decrease. *J. Biomed. Opt.* **16**, 117002 (2011).

62. V. Backman, V. Gopal, M. Kalashnikov, K. Badizadegan, R. Gurjar, A. Wax, I. Georgakoudi, M. Mueller, C. W. Boone, R. R. Dasari, M. S. Feld, Measuring cellular structure at submicrometer scale with light scattering spectroscopy. *IEEE J. Sel. Top.Quantum Electron.* **7**, 887–893 (2001).

63. E. T. Jelly, Z. A. Steelman, H. Zhang, K. K. Chu, C. C. Cotton, S. Eluri, N. J. Shaheen, A. Wax, Next-generation endoscopic probe for detection of esophageal dysplasia using combined OCT and angle-resolved low-coherence interferometry. *Biomed. Opt. Express* **15**, 1943–1958 (2024).

64. C. van Dooijeweert, P. J. van Diest, I. O. Ellis, Grading of invasive breast carcinoma: The way forward. *Virchows Arch.* **480**, 33–43 (2022).

65. S. C. Wetstein, V. M. T. de Jong, N. Stathonikos, M. Opdam, G. M. H. E. Dackus, J. P. W. Pluim, P. J. van Diest, M. Veta, Deep learning-based breast cancer grading and survival analysis on whole-slide histopathology images. *Sci. Rep.* **12**, 15102 (2022).

66. R. C. Chan, C. K. C. To, K. C. T. Cheng, T. Yoshikazu, L. L. A. Yan, G. M. Tse, Artificial intelligence in breast cancer histopathology. *Histopathology* **82**, 198–210 (2023).

67. G. Xi, W. Guo, D. Kang, J. Ma, F. Fu, L. Qiu, L. Zheng, J. He, N. Fang, J. Chen, J. Li, S. Zhuo, X. Liao, H. Tu, L. Li, Q. Zhang, C. Wang, S. A. Boppart, J. Chen, Large-scale tumor-associated collagen signatures identify high-risk breast cancer patients. *Theranostics* **11**, 3229–3243 (2021).

68. S. Gesta, Y.-H. Tseng, C. R. Kahn, Developmental origin of fat: Tracking obesity to its source. *Cell* **131**, 242–256 (2007).

69. L. X. Ma, C. C. Wang, J. Y. Tan, Light scattering by densely packed optically soft particle systems, with consideration of the particle agglomeration and dependent scattering. *Appl. Opt.* **58**, 7336–7345 (2019).

70. J. M. Schmitt, G. Kumar, Optical scattering properties of soft tissue: A discrete particle model. *Appl. Optics* **37**, 2788–2797 (1998).

71. S. J. Kirkpatrick, D. D. Duncan, E. M. Wells-Gray, Detrimental effects of speckle-pixel size matching in laser speckle contrast imaging. *Opt. Lett.* **33**, 2886–2888 (2008).

72. L. Kaufman, P. J. Rousseeuw, *Finding Groups in Data: An Introduction to Cluster Analysis* (John Wiley & Sons, 2009).

73. P. J. Rousseeuw, Silhouettes: A graphical aid to the interpretation and validation of cluster analysis. *J. Comput. Appl. Math.* **20**, 53–65 (1987).

74. N. Uribe-Patarroyo, A. L. Post, S. Ruiz-Lopera, D. J. Faber, B. E. Bouma, Noise and bias in optical coherence tomography intensity signal decorrelation. *OSA Contin.* **3**, 709–741 (2020).

75. J. C. Ramella-Roman, S. A. Prahl, S. L. Jacques, Three Monte Carlo programs of polarized light transport into scattering media: Part I. *Opt. Express* **13**, 10392–10405 (2005).

76. J. C. Ramella-Roman, S. A. Prahl, S. L. Jacques, Three Monte Carlo programs of polarized light transport into scattering media: Part II. *Opt. Express* **13**, 10392–10405 (2005).
